# Transcriptomic Profiling Identifies Candidate Genes Involved in the Salt Tolerance of the Xerophyte *Pugionium cornutum*

**DOI:** 10.3390/genes10121039

**Published:** 2019-12-12

**Authors:** Yan-Nong Cui, Fang-Zhen Wang, Cheng-Hang Yang, Jian-Zhen Yuan, Huan Guo, Jin-Lin Zhang, Suo-Min Wang, Qing Ma

**Affiliations:** Key Laboratory of Grassland Livestock Industry Innovation, Ministry of Agriculture and Rural Affairs, State Key Laboratory of Grassland Agro-ecosystems, College of Pastoral Agriculture Science and Technology, Lanzhou University, Lanzhou 730020, China; cuiyn17@lzu.edu.cn (Y.-N.C.); wangfzh18@lzu.edu.cn (F.-Z.W.); 13557012949@163.com (C.-H.Y.); yuanjzh18@lzu.edu.cn (J.-Z.Y.); Guoh16@lzu.edu.cn (H.G.); jlzhang@lzu.edu.cn (J.-L.Z.); smwang@lzu.edu.cn (S.-M.W.)

**Keywords:** salt tolerance, transcriptome, differentially expressed genes, xerophyte, *Pugionium cornutum*

## Abstract

The xerophyte *Pugionium cornutum* adapts to salt stress by accumulating inorganic ions (e.g., Cl^−^) for osmotic adjustment and enhancing the activity of antioxidant enzymes, but the associated molecular basis remains unclear. In this study, we first found that *P. cornutum* could also maintain cell membrane stability due to its prominent ROS-scavenging ability and exhibits efficient carbon assimilation capacity under salt stress. Then, the candidate genes associated with the important physiological traits of the salt tolerance of *P. cornutum* were identified through transcriptomic analysis. The results showed that after 50 mM NaCl treatment for 6 or 24 h, multiple genes encoding proteins facilitating Cl^−^ accumulation and NO_3_^−^ homeostasis, as well as the transport of other major inorganic osmoticums, were significantly upregulated in roots and shoots, which should be favorable for enhancing osmotic adjustment capacity and maintaining the uptake and transport of nutrient elements; a large number of genes related to ROS-scavenging pathways were also significantly upregulated, which might be beneficial for mitigating salt-induced oxidative damage to the cells. Meanwhile, many genes encoding components of the photosynthetic electron transport pathway and carbon fixation enzymes were significantly upregulated in shoots, possibly resulting in high carbon assimilation efficiency in *P. cornutum*. Additionally, numerous salt-inducible transcription factor genes that probably regulate the abovementioned processes were found. This work lays a preliminary foundation for clarifying the molecular mechanism underlying the adaptation of xerophytes to harsh environments.

## 1. Introduction

Soil salinity is one of the primary abiotic factors that limits the sustainable development of agriculture worldwide, and approximately one fifth of the world’s cultivated land has been affected by salinity [1]. Salt tolerance in plants is a complex trait involving responses to cellular osmotic and ionic stresses and their consequent secondary stresses (e.g., oxidative stress), which are all polygenic processes [2]. Most conventional and staple crop and forage species are very sensitive to high concentrations of salt in soils and have limited genetic potential for salt tolerance because of their long-term growth under favorable cultivation conditions [2]. The breeding of crops with high yield and quality under saline conditions by using molecular genetics is an effective strategy to promote food security [3]. Despite the abundant molecular knowledge derived from model plants, such as *Arabidopsis thaliana* and *Oryza sativa*, their low stress tolerance limits the usefulness of these species in current breeding programs [4]. Certain wild plant species, including halophytes and xerophytes, however, have evolved multiple protective mechanisms to successfully thrive in extremely harsh environments and, as a result, harbor prominent abiotic stress tolerance genes [4,5]. Hence, it is possible to genetically improve salt tolerance in crops and forage through an improved understanding of the molecular basis underlying the strategies for adaptation to soil salinity employed by these halophytes and xerophytes [6].

*P. cornutum*, a xerophytic desert plant in the genus *Pugionium* Gaertn belonging to Brassicaceae, is primarily found in arid and semiarid regions of northwestern China and exhibits strong adaptability to various environmental stresses including salinity and drought [7]. In local areas, this species plays an important role in sand fixation and soil and water conservation due to its well-developed root systems; moreover, its favorable medicinal properties and nutrient content make this plant attractive as a traditional Chinese herb and vegetable [8]. Previous studies have found increased activity of enzymes involved in reactive oxygen species (ROS) scavenging, such as superoxide dismutase (SOD), catalase (CAT), and peroxidase (POD), in *P. cornutum* under salt and drought stress [9,10]. It was also demonstrated that *P. cornutum* is a typical Cl^−^-tolerant species and can tolerate high tissue Cl^−^ concentrations that are even toxic to some other Cl^−^-tolerant species; moreover, *P. cornutum* can directly use Cl^−^ as an efficient and beneficial osmoticum for enhancing leaf turgor and hydration under salt stress [11,12]. In addition, substantially increased accumulation of other major inorganic osmoticums, such as Na^+^, SO_4_^2−^, and PO_4_^3−^, as well as relatively stable concentrations of K^+^ and NO_3_^−^, were observed in the roots and shoots of *P. cornutum* under NaCl treatment [11,12]. Therefore, the substantial absorption of inorganic osmoticums, especially Cl^−^, and the maintenance of K^+^ and NO_3_^−^ homeostasis are vital physiological mechanisms in the adaptation of *P. cornutum* to salt stress. Although these physiological traits of the salt tolerance of *P. cornutum* have been documented, other physiological responses, such as photosynthetic performance, and in particular, the molecular mechanisms underlying the physiological traits remain elusive. Furthermore, studies on the molecular mechanisms involved in the ability of higher plants to tolerate Cl^−^ toxicity continue to lag behind and have mainly focused on the model plant *A. thaliana* and Cl^−^-sensitive crops such as soybean, citrus, and grape [13]. Thus, understanding the molecular basis of *P. cornutum* coping with Cl^−^ toxicity would help to further elucidate Cl^−^ tolerance in higher plants.

RNA sequencing (RNA-Seq) is a rapid and cost-effective method for both mapping and quantifying the transcriptome in eukaryotes and is widely used to analyze gene expression in plants at specific developmental stages or under specific physiological conditions, providing substantial insight into the molecular processes involved in plants responses to abiotic stress [14]. In the present work, we investigated the physiological indexes related to plant growth, the stability of the cell membrane, and the photosynthesis of *P. cornutum* under different NaCl treatments, and then generated a transcriptomic dataset for studying the molecular mechanisms of salt tolerance in *P. cornutum* using Illumina high-throughput sequencing technology. Then, we analyzed the differentially expressed functional genes related to ion (especially Cl^−^) transport, ROS scavenging, and photosynthesis, as well as transcription factor (TF) genes under 50 mM NaCl treatment by using a tag-based digital gene expression (DGE) profiling technique.

## 2. Materials and Methods

### 2.1. Plant Growth Conditions and Salt Treatments in Sand Culture Experiments

In the present study, seeds of *P. cornutum* were collected from plants grown in Mu Us Sandland in the Inner Mongolia Autonomous Region, China. A corresponding voucher specimen (No. Q.S. Yu, 6065) has been deposited in the Herbarium of the School of Life Science, Lanzhou University [7]. After removal of the bracts, seeds were disinfected with 75% alcohol for 30 s, sterilized with 5% NaClO for 10 min, rinsed six times with distilled water, soaked in distilled water for one day, and then germinated at 28 °C in the dark. After approximately five days, the seedlings were transplanted into plastic pots (one seedling/pot) filled with coarse-grained silica sand and irrigated with Hoagland solution containing 2 mM KNO_3_, 0.5 mM KH_2_PO_4_, 0.5 mM MgSO_4_, 0.5 mM Ca(NO_3_)_2_, 60 µM Fe-citrate, 50 µM H_3_BO_3_, 10 µM MnCl_2_, 1.6 µM ZnSO_4_, 0.6 µM CuSO_4_, and 0.05 µM Na_2_MoO_4_ (the pH was adjusted to 5.7 by using 1 M Tris). This sand culture system is very similar to the hydroponic culture system because the plant roots in both systems come in direct contact the culture or treatment solution, which allows rapid cleaning of the roots and harvesting of intact root samples in a timely manner, especially for subsequent transcriptome sequencing. The solution was refreshed every three days. All seedlings were grown in a greenhouse, where the daily photoperiod was 16 h/8 h (light/dark), the temperature was 28 °C/23 °C (day/night), the light flux density was approximately 500 µmol m^−2^ s^−1^, and the relative humidity was approximately 60%.

After one month, uniform seedlings were treated with Hoagland nutrient solution supplemented with 0 (control), 20, 50, or 100 mM NaCl. For the 100 mM NaCl treatment, plants were treated with 50 mM NaCl for one day and were then transferred to the 100 mM NaCl treatment. Plants were harvested at seven days after treatments for physiological analysis. The treatment solutions were changed once every two days to maintain a constant NaCl concentration.

### 2.2. Determination of the Physiological Indices Related to Plant Growth, Stability of the Cell Membrane, and Photosynthesis

Roots and shoots of individual seedlings were gently separated, and then, the fresh weight (FW) of the roots and shoots was immediately measured. Subsequently, all samples were oven-dried at 80 °C for three days for dry weight (DW) measurements.

The leaf relative membrane permeability (RMP) was assessed as described by Yue et al. [15] using a conductivity meter (EC215, HANNA instruments, Padovana, Italy). Fresh leaves soaked in deionized water were first shaken gently at 25 °C for 2 h to measure the leaked electrolytes (E1); then, the leaves were incubated in a boiling water bath for 1 h to measure the total electrolytes (E2). Leaf RMP (%) = E1/E2 × 100. The malonedialdehyde (MDA) in the fresh leaves was extracted by using 10% (*w/v*) trichloroacetic acid, and the MDA concentration was determined according to the method described by Yue et al. [15] using a UV spectrophotometer (UV-2102C, Unico Instrument Co., Ltd., Shanghai, China).

An open infrared portable gas exchange fluorescence system (GFS-3000, Heinz Walz GmbH, Effeltrich, Germany) was used to measure the net photosynthesis rate (Pn) and stomatal conductance (Gs). Parameters were measured in fully expanded mature leaves from 9:00 a.m. to 11:30 a.m. in the greenhouse. During measurement, the temperature, relative air humidity, photosynthetic photon flux density and CO_2_ concentration in the leaf chamber of the apparatus were constantly maintained at 25 °C, 50%, 1000 ± 50 μmol m^−2^ s^−1^, and 420 ± 20 μmol mol^−1^, respectively. Chlorophyll was extracted from the fresh leaf samples with 80% acetone and 95% ethanol (*v/v* = 1:1). Then, the chlorophyll a and chlorophyll b content was estimated with a UV spectrophotometer as described by Ma et al. [16].

The results for the abovementioned parameters are all presented as the means with standard errors (n = 6). All the data were subjected to one-way analysis of variance (ANOVA) using SPSS 19.0 (SPSS Inc., Chicago, IL, USA). Duncan’s multiple range tests were used to detect significant differences between means at a significance level of *p* < 0.05.

### 2.3. Plant Growth Conditions and Treatments for Transcriptome Sequencing

The plant growth conditions were the same as those described in Section 2.1. After one month, uniform seedlings were randomly divided into two groups: a control group (C) and a salt treatment group (S). In the control group, seedlings were irrigated with normal Hoagland solution; after 6 and 24 h, root (R) and shoot (S) samples were collected and labeled as C6R, C24R, C6S, and C24S, respectively. In the salt group, seedlings were treated with Hoagland solution containing 50 mM NaCl; after 6 and 24 h, root (R) and shoot (S) samples were collected and labeled as S6R, S24R, S6S, and S24S, respectively. Each sample was collected from five or six individual seedlings, and immediately frozen in liquid nitrogen for total RNA extraction.

### 2.4. Transcriptome Sequencing

Total RNA was extracted from the four root samples (C6R, C24R, S6R, S24R) and four shoot samples (C6S, C24S, S6S, S24S) using the RNeasy Plant Mini Kit (Qiagen, Venlo, The Netherlands). Then, residual DNA was removed from the extracted RNA by treatment with DNase I at 37 °C for 40 min. RNA integrity and purity were determined using a NanoDrop ND 1000 instrument (Thermo Scientific, Waltham, MA, USA).

To obtain the transcriptomes of the roots and shoots of *P. cornutum*, the four RNA samples from the roots were pooled together and the four RNA samples from the shoots were pooled together. Poly(A) mRNA from the two pooled RNA samples was purified and then fragmented into small pieces as described by Dang et al. [17]. The first-strand cDNA and second-strand cDNA of the mRNA fragments were synthesized for end repair and poly(A) addition and then linked to sequencing adapters according to Dang et al. [17]. Finally, we constructed two paired-end cDNA libraries (one for the roots and one for the shoots) and sequenced the cDNA in each library on an Illumina HiSeq™ 2000 platform at the Beijing Genomics Institute (BGI, Shenzhen, China). The raw data have been deposited in the NCBI Sequence Read Archive (PRJNA512400).

The raw sequencing reads were then filtered to obtain high-quality clean reads. Subsequently, transcriptome de novo assembly and unigene acquisition were performed. Finally, the obtained unigenes were clustered into the following two classes: (i) clusters: each cluster contained several unigenes sharing ≥70% sequence similarity with each other and was named with “CL + cluster digital ID” as the prefix followed by the contig number (e.g., CL1. Contig 1, 2, 3…); (ii) singletons: each singleton represented one individual sequence that did not reach 70% similarity with any other sequence or fall into any assembly and was named with “Unigene” as the prefix followed by the gene digital ID (e.g., Unigene 1, 2, 3…). To obtain protein functional annotations, all the unigene sequences were aligned against protein databases, including the non-redundant protein (NR) database, the nonredundant nucleotide (NT) database, Swiss-Prot, Kyoto Encyclopedia of Genes and Genomes (KEGG), Clusters of Orthologous Groups (COG), and Gene Ontology (GO), using BLASTX with a significance threshold of *E*-value < 10^−5^.

### 2.5. Differentially Expressed Genes Analysis

The eight total RNA samples extracted from the *P. cornutum* tissue samples were used to construct eight independent tagged sequencing libraries (C6R, C24R, S6R, S24R, C6S, C24S, S6S, and S24S) in parallel with the tag-based digital gene expression (DGE) system as described by Ma et al. [6]. Then, each tagged sequencing library was sequenced on an Illumina HiSeq™ 2000 platform at the BGI.

The obtained tags in each library were filtered as described by Ma et al. [6]. Then, the clean tags in each cDNA library were mapped to the all-unigenes data from the transcriptome sequencing using the short reads alignment program SOAPaligner/soap2 to determine the protein functional annotations of the genes. The transcript abundance of each gene was determined by the reads per kilobase of exon model per million mapped read (RPKM) method [18].

To identify genes responding to salt treatment, the log_2_ ratio of gene transcript abundance between tagged sequencing libraries for the salt-treated tissue and the corresponding untreated tissue (S6R vs. C6R, S24R vs. C24R, S6S vs. C6S, S24S vs. C24S) was calculated. The statistical significance of the differential expression level for each gene was determined by evaluating the probability (*p*-value) of a gene being transcribed equally between two tagged libraries using the formula proposed by Audic et al. [19]. The results of all statistical tests were corrected for multiple tests, and the false discovery rate (FDR) was used to determine the threshold *p*-value in multiple tests [6]. In this study, a gene with FDR < 0.001 and absolute value of log_2_ ratio > 1 was termed a differentially expressed gene (DEG). Finally, the upregulated and downregulated DEGs related to ion transport, the ROS-scavenging system, photosynthesis, and transcriptional factors were analyzed in roots and shoots.

### 2.6. Real-Time Quantitative PCR Validation of DEG Results

An independent real-time quantitative PCR (qPCR) experiment was conducted to validate the DEG results from RNA sequencing (RNA-seq). *P. cornutum* seedlings were cultured and treated in exactly the same manner as described in Section 2.3; then, root and shoot samples were harvested for qRT-PCR validation. We randomly selected 20 DEGs from roots and 20 DEGs from shoots under both 6 and 24 h of salt treatments from our RNA-seq data. Then, the changes in expression of these DEGs under 50 mM NaCl treatment relative to the control condition were investigated using the qPCR method as described by Guo et al. [20], where *ACTIN2* served as an internal reference. Subsequently, a correlation analysis between the data obtained by RNA-seq and qPCR was performed following Guo et al. [20].

## 3. Results

### 3.1. Changes in Plant Growth, Cell Membrane Stability, and Photosynthesis Performance of P. cornutum under Different NaCl Treatments

**Figure 1:**
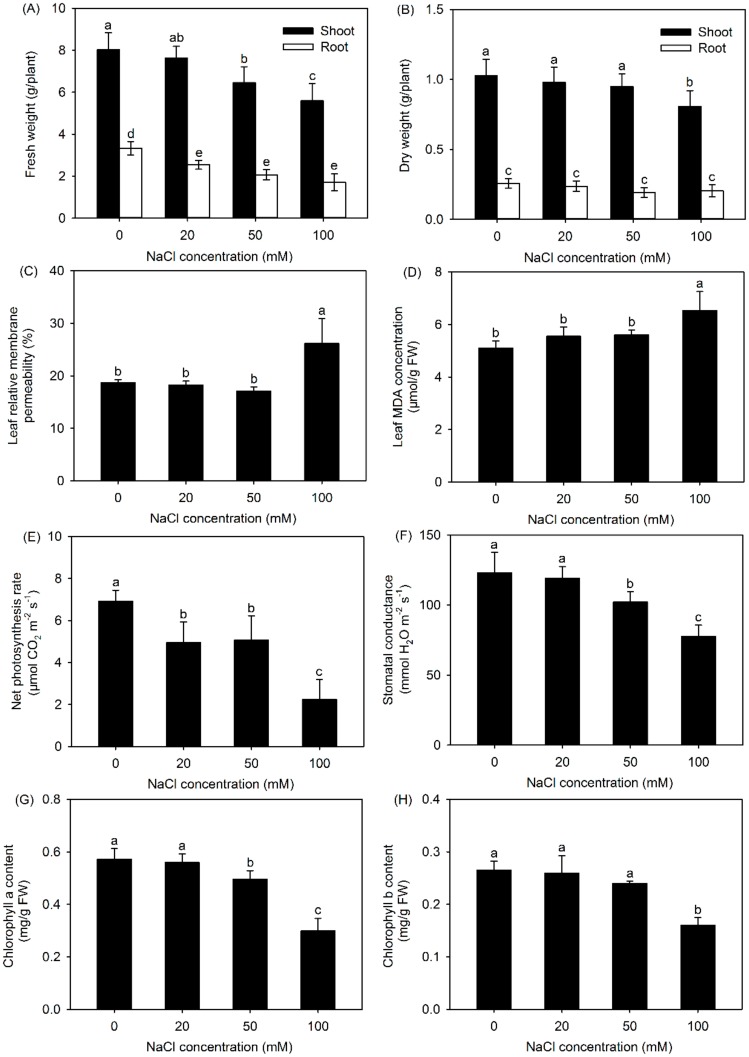
Fresh weight (FW) (**A**), dry weight (DW) (**B**), leaf relative membrane permeability (RMP) (**C**), leaf malondialdehyde (MDA) concentration (**D**), net photosynthesis rate (Pn) (**E**), stomatal conductance (Gs) (**F**), chlorophyll a content (**G**), and chlorophyll b content (**H**) of *P. cornutum* exposed to 0, 20, 50, and 100 mM NaCl for seven days. Values are the means ± SDs (n = 6). Columns with different lowercase letters are significantly different at *p* < 0.05 (Duncan’s test).

Compared with the control (no additional NaCl), treatment with NaCl, especially 100 mM NaCl, significantly decreased the shoot and root fresh weight (FW) of *P. cornutum*, as seen in Figure 1A. It was observed that the shoot and root dry weight (DW) was unaffected by the 20 and 50 mM NaCl treatments, while shoot DW was significantly decreased in the presence of 100 mM NaCl, as seen in Figure 1B, compared with the values under the control condition, indicating that external NaCl at lower than 50 mM had no adverse effect on the dry biomass of *P. cornutum*.

The addition of 100 mM NaCl significantly elevated the leaf RMP and MDA concentration; however, both of these parameters remained unchanged when plants were exposed to 20 and 50 mM NaCl, as seen in Figure 1C,D. Salt stress induces an increase in the levels of ROS, which will destroy cell membranes by lipid peroxidation, resulting in increase of leaf RMP, and MDA is an end product of membrane lipid peroxidation [15,21]. The unchanged leaf RMP and MDA concentrations, as seen in Figure 1C,D, and increased activity of antioxidant enzymes [9,10] represented that *P. cornutum* could maintain cell membrane stability due to its prominent ROS-scavenging ability under external NaCl concentrations lower than 50 mM.

The leaf net Pn and Gs under all the NaCl treatments (except for the Gs under 20 mM NaCl treatment) were significantly reduced compared with those under the control condition, as seen in Figure 1E,F. Although the leaf chlorophyll a content under 50 mM treatment was reduced by approximately 10% compared with that under the control condition, the leaf chlorophyll b content was unaltered, as seen in Figure 1G,H, indicating that chlorophyll biosynthesis in *P. cornutum* exposed to 50 mM NaCl treatment remained relatively stable. Given that the dry biomass of *P. cornutum* remained unchanged under 50 mM NaCl treatment, as seen in Figure 1B, *P. cornutum* could maintain a high carbon assimilation efficiency under the external NaCl lower than 50 mM.

All these results suggested that *P. cornutum* could tolerate the 50 mM NaCl treatment in the sand culture system. In contrast, 50 mM NaCl severely impaired the growth of the glycophytes, such as Arabidopsis and even the salt-tolerant rice cultivar in hydroponics [22,23], suggesting that under 50 mM NaCl treatment, *P. cornutum* already exhibits a much more effective genetic response network to maintain normal growth, compared to glycophytes. Therefore, in the subsequent transcriptomic analysis, we chose 50 mM NaCl for salt treatment of *P. cornutum* seedlings.

### 3.2. Transcriptome Sequencing, De Novo Assembly, and Functional Annotation

Transcriptome sequencing generated more than 119 and 118 million raw reads from the roots and shoots of *P. cornutum*, respectively, as seen in Supplementary Table S1. After exclusion of the low-quality reads, including empty reads, adapter reads, and reads with unknown “N” nucleotide or only one copy number, 117 and 116 million clean reads with GC percentages of 45% and 46% were obtained from the roots and shoots, respectively, as seen in Table S1. The total clean read/total raw read percentages were more than 98% for both roots and shoots. Only a small number of reads were excluded by filtering, indicating that the data were highly reliable.

The de novo assembly of total clean reads generated 64,978 unigenes from the shoots and 80,307 unigenes from the roots, with an average length of 1091 and 997 bp, respectively, as seen in Table 1. After further assembly and redundancy elimination of these unigenes using CAP Assembler, 72,068 gap-free unigenes with an average lengths of 1243 bp were obtained, as seen in Table 1. As shown in Supplementary Figure S1, the length of 34,108 unigenes (more than 50%) were greater than 1000 bp.

In total, 63,396 unigenes were matched to known homologs from other plant species by alignment against protein databases, including NR, NT, Swiss-Prot, KEGG, COG, and GO, accounting for 88% of the total unigenes, as seen in Supplementary Table S2. Specifically, 57,695, 60,628, 39,893, 35,136, 24,608, and 53,435 unigenes had functional annotations in the above six databases, accounting for 80%, 84%, 55%, 49%, 34%, and 74% of the total unigenes, respectively, as seen in Table S2.

### 3.3. Identification of DEGs in the Roots and Shoots of P. cornutum Treated with 50 mM NaCl

**Figure 2:**
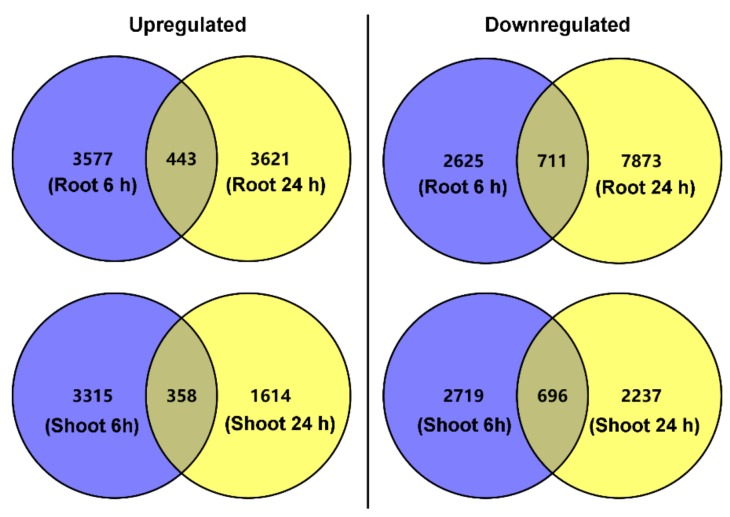
Venn diagrams showing the total number of differentially expressed genes (DEGs) in roots and shoots of *P. cornutum* under 50 mM NaCl treatment for 6 and 24 h. The blue circles represent the number of upregulated or downregulated DEGs in roots or shoots exclusively under 50 mM NaCl treatment for 6 h. The yellow circles represent the number of upregulated or downregulated DEGs in roots or shoots exclusively under 50 mM NaCl treatment for 24 h. The overlaps between the blue circles and yellow circles represent the number of upregulated or downregulated DEGs in roots or shoots under 50 mM NaCl treatment for both 6 and 24 h.

The DEGs in the roots and shoots were then identified. In total, 3577 DEGs in roots and 3315 DEGs in shoots were upregulated exclusively after salt treatment (50 mM NaCl) for 6 h, as seen in Figure 2. After salt treatment for 24 h, another 3621 and 1614 upregulated DEGs in roots and shoots were observed, respectively, as seen in Figure 2. Notably, 443 DEGs in roots and 358 DEGs in shoots were upregulated after salt treatment for both 6 and 24 h. In total, 2625 DEGs in roots and 2719 DEGs in shoots were downregulated exclusively after salt treatment for 6 h, and 7873 DEGs in roots and 2237 DEGs in shoots were downregulated exclusively after salt treatment for 24 h, as seen in Figure 2; meanwhile, there were 711 and 696 downregulated DEGs in roots and shoots, respectively, after salt treatment for both 6 and 24 h, as seen in Figure 2.

### 3.4. DEGs Related to Ion Transport

The absorption and accumulation of inorganic osmoticums, such as Cl^−^, Na^+^, SO_4_^2−^, and PO_4_^3−^, as well as maintenance of the homeostasis of the inorganic macronutrients K^+^ and NO_3_^−^, are primary physiological mechanisms in the salt tolerance of *P. cornutum* [11,12]. Therefore, we first analyzed the effects of NaCl treatment on the transcript levels of DEGs related to ion transport in the roots and shoots of *P. cornutum*.

**Figure 3:**
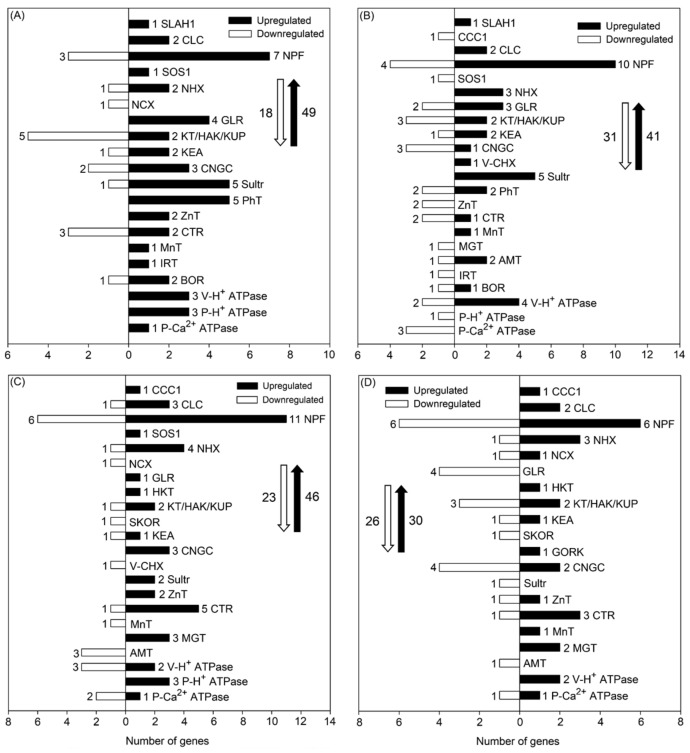
Number of DEGs related to ion transport under 50 mM NaCl treatment for 6 and 24 h in roots ((**A**,**B**), respectively) and in shoots ((**C**,**D**), respectively) of *P. cornutum*. The white downward arrows and black upward arrows show the total number of downregulated DEGs and upregulated DEGs, respectively.

After 50 mM NaCl treatment for 6 and 24 h, 49 and 41 DEGs, respectively, associated with Cl^−^, NO_3_^−^, Na^+^, K^+^, Ca^2+^, SO_4_^2−^, PO_4_^3−^, Zn^2+^, Cu^2+^, NH_4_^+^, Mn^2+^, Mg^2+^, Fe^2+^, BO_3_^3−^, and H^+^ transport were upregulated in roots, and 18 and 31 DEGs, respectively, were downregulated in roots, as seen in Figure 3A,B. Among the upregulated DEGs, a large proportion were Cl^−^ and/or NO_3_^−^ transporter-encoding genes, including the slow-type anion channel-associated homolog 1-encoding gene (*SLAH1*), chloride channel-encoding genes (*CLCs*), and nitrate transporter 1/peptide transporter-encoding genes (*NPFs*). Notably, the transcript levels of *SLAH1*, *CLCg*, and *NPF6.4* were upregulated in roots after 50 mM NaCl treatment for both 6 and 24 h, as seen in Table 2 and Supplementary Tables S3 and S4, indicating that these genes might be closely involved in Cl^−^ absorption and accumulation as well as NO_3_^−^ homeostasis in *P. cornutum* under saline conditions. Furthermore, it was observed that *SLAH1* was not expressed at all in roots under the control condition (the RPKM value of *SLAH1* was 0.01 under the control condition), while its expression level was increased more than eight-fold after salt treatment for both 6 and 24 h, as seen in Tables S3 and S4, indicative of a vital role of *SLAH1* in the salt response of *P. cornutum*. Among the downregulated DEGs related to Cl^−^ and/or NO_3_^−^ transport, the transcript abundance of the cation-chloride cotransporter 1-encoding gene (*CCC1*) was substantially decreased in roots under salt treatment for 24 h, as seen in Table S4. Meanwhile, the transcript abundance of the gene encoding the plasma membrane Na^+^/H^+^ antiporter SOS1 in roots was upregulated after salt treatment for 6 h, but downregulated after salt treatment for 24 h, as seen in Figure 3A,B and Tables S3 and S4, indicating that *SOS1* in roots might regulate salt tolerance in *P. cornutum* during relatively short-term salt treatment. The transcript abundance of the tonoplast Na^+^/H^+^ antiporter-encoding gene *NHX1* was upregulated in roots after 24 h of salt treatment, and the Golgi-located Na^+^/H^+^ antiporter-encoding genes *NHX5* and *NHX6* were upregulated after both 6 and 24 h of salt treatment, as seen in Figure 3A,B and Tables S3 and S4. These three *NHX* genes likely facilitate Na^+^/K^+^ homeostasis in roots of *P. cornutum* under saline conditions. Furthermore, several K^+^ transporter-encoding genes that also contribute to Na^+^/K^+^ homeostasis, including members of the K^+^ transporter gene family (e.g., *KT2*, *KT3*, *KUP2*, *HAK3*, *KEA1*, *KEA4*, *KEA5*, and *KEA6*), were upregulated under salt treatment for either 6 or 24 h, as seen in Figure 3A,B and Tables S3 and S4. Many cyclic nucleotide-gated channel-encoding genes (*CNGCs*), glutamate receptor-encoding genes (*GLRs*), and Na^+^/Ca^2+^ exchanger-encoding genes (*NCX*s) that are involved in K^+^, Na^+^, and Ca^2+^ transport in the roots of *P. cornutum* were upregulated under salt treatment, as seen in Figure 3A,B. Many upregulated DEGs related to the transport of other inorganic ions, including SO_4_^2−^ (e.g., *Sultr1.2*, *Sultr2.1*, *Sultr3.1*), PO_4_^3−^ (e.g., *PhT1.3*, *PhT2.1*, *PhT4.3*, *PHO1*), Zn^2+^ (e.g., *ZnT1*, *ZnT8*, *ZnT12*), Cu^2+^ (e.g., *CTR2*, *CTR5*, *CCH*, *PAA1*), Mn^2+^ (e.g., *PDR2*), Fe^2+^ (e.g., *IRT1*), Mg^2+^ (e.g., *MgT*), NH_4_^+^ (e.g., *AMT1.1*, *AMT1.2*, *AMT2*), and BO_3_^3−^ (e.g., *BOR3*, *BOR4*), were also identified in the roots of *P. cornutum* under either 6 or 24 h of salt treatment, as seen in Figure 3A,B and Tables S3 and S4. Additionally, as the transport of inorganic ions across cell membranes is generally coupled with H^+^ as the proton motive force [24], the upregulated plasma membrane H^+^ ATPase-encoding gene (*P-H^+^ ATPase*) and vacuolar H^+^ ATPase-encoding gene (*V-H^+^ ATPase*) in roots under salt treatment identified by our data, as seen in Figure 3A,B, likely play essential roles in ion transport across the root cell plasma membrane and tonoplast, in addition to maintaining the cellular membrane electrochemical gradient.

After 50 mM NaCl treatment for 6 and 24 h, 46 and 30 DEGs associated with inorganic ion transport were upregulated, respectively, and 23 and 26 DEGs were downregulated, respectively, in shoots, as seen in Figure 3C,D. In contrast to the observation in roots, the transcript level of *CCC1* in shoots was considerably upregulated under both 6 and 24 h of salt treatment, as seen in Figure 3C,D and Supplementary Tables S5 and S6. The transcript abundance of *CLCg* in shoots was upregulated under salt treatment for both 6 and 24 h, as seen in Table 3, and several *NPFs*, such as *NPF6.3* and *NPF6.4*, which encode NO_3_^−^ and/or Cl^−^ transporters, were also upregulated under salt treatment, as seen in Tables S5 and S6. These genes might also be essential for Cl^−^ accumulation and NO_3_^−^ homeostasis in shoots of *P. cornutum* under saline conditions. Certain DEGs encoding Na^+^/H^+^ antiporters (SOS1 and NHX1), K^+^ transporters (KT2, KUP2, KEA1, KEA4, KEA6), cation channels (CNGC1 and CNGC6), and glutamate receptors (GLR3.3) were upregulated in shoots after salt treatment for either 6 or 24 h, as seen in Tables S5 and S6, indicating that these genes might also regulate Na^+^/K^+^ homeostasis in shoots of *P. cornutum* under saline conditions. Furthermore, several DEGs related to Na^+^ or K^+^ transport, including the high-affinity K^+^ transporter-encoding gene (*HKT1*), stelar K^+^ outward rectifying channel-encoding gene (*SKOR*), and guard cell outward-rectifying K^+^ channel-encoding gene (*GORK*), were found in shoots, but not in roots, after salt treatment, as seen in Figure 3. Many DEGs related to the transport of other inorganic nutrients or osmoticums, such as SO_4_^2−^, Zn^2+^, Cu^2+^, Mn^2+^, Fe^2+^, Mg^2+^, and NH_4_^+^, were identified in shoots after salt treatment for either 6 or 24 h, as seen in Figure 3C,D, indicating that these genes might also regulate osmotic adjustment of *P. cornutum* in the shoots under saline conditions. Among the DEGs encoding V-H^+^ ATPase, P-H^+^ ATPase, and plasma membrane Ca^2+^ ATPase (P-Ca^2+^ ATPase) in shoots, *V-H^+^ ATPase c2*, *V-H^+^ ATPase e1*, and *V-H^+^ ATPase h* were not expressed under the control condition but were highly expressed under salt treatment, as seen in Tables S5 and S6, suggesting that these three genes might play vital roles in vacuole compartmentation of inorganic osmoticums for facilitating shoot osmotic adjustment.

### 3.5. DEGs Related to the ROS-Scavenging System

Maintaining cell membrane stability, as seen in Figure 1C,D, due to its prominent ROS-scavenging ability [9,10] is one of the major physiological traits related to the salt tolerance of *P. cornutum*. The ROS-scavenging system in higher plants mainly consists of the ascorbate–glutathione (AsA–GSH) cycle, glutathione peroxidase (GPX) pathway, catalase (CAT) pathway, and peroxiredoxin/thioredoxin (PrxR/Trx) pathway [6]. Therefore, the DEGs related to the above pathways were analyzed.

**Figure 4:**
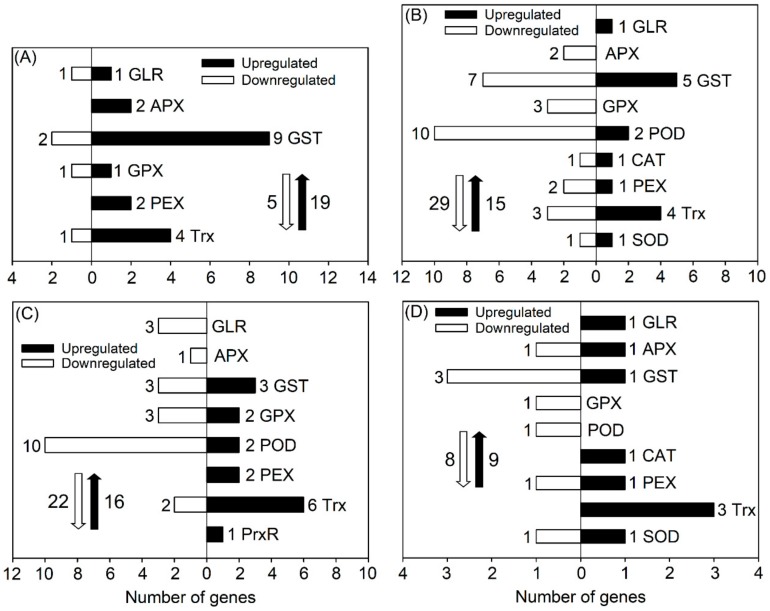
Number of DEGs related to the reactive oxygen species (ROS) scavenging system under 50 mM NaCl treatment for 6 and 24 h in roots ((**A**,**B**), respectively) and in shoots ((**C**,**D**), respectively) of *P. cornutum*. Glutamate receptor (GLR), ascorbate peroxidase (APX), and glutathione S-transferase (GST) are involved in the ascorbate–glutathione (AsA–GSH) cycle; GST, glutathione peroxidase (GPX), and peroxidase (POD) are involved in the GPX pathway; catalase (CAT) and peroxisomal biogenesis factor (PEX) are involved in the CAT pathway; and thioredoxin (Trx) and peroxiredoxin (PrxR) are involved in the PrxR/Trx pathway. The white downward and black upward arrows show the total number of downregulated DEGs and upregulated DEGs, respectively.

After salt treatment (50 mM) for 6 and 24 h, a total of 19 and 15 DEGs, respectively, categorized in the abovementioned ROS-scavenging pathways in the roots of *P. cornutum* were upregulated, and five and twenty-nine DEGs, respectively, were downregulated, as seen in Figure 4A,B. Among the upregulated DEGs in roots after 6 h of salt treatment, most were glutathione S-transferase-encoding genes (*GSTs*) that are involved in both the ASA-GSH and GPX pathways, and thioredoxin-encoding genes (*Trxs*) that are involved in the PrxR/Trx pathway, as seen in Figure 4A). Hence, the ASA-GSH, GPX and PrxR/Trx pathways might be the major components of the root ROS-scavenging system for *P. cornutum* in adaptation to salinity. After 24 h of salt treatment, although many DEGs associated with ROS scavenging were downregulated, the transcript abundances of *GST-U10* (no expression under the control condition) and *TrxH8* were still clearly upregulated, as seen in Figure 4B and Supplementary Table S8, indicating that these two genes might play vital roles in ROS scavenging in the roots of *P. cornutum* under saline conditions. Furthermore, after salt treatment for 24 h, two upregulated peroxidase-encoding genes (*PODs*), one upregulated catalase-encoding gene (*CAT*), and one upregulated superoxide dismutase-encoding gene (*SOD*) emerged, as seen in Figure 4B. These genes probably facilitate root ROS scavenging in *P. cornutum* during relatively long-term salt treatment.

Following 6 h of 50 mM NaCl treatment, the expression levels of three *GST* genes and two *GPX* genes in the GPX pathway were upregulated in *P. cornutum* shoots, as seen in Figure 4C. In particular, *GST-F9* showed no expression under the control condition, but high expression was detected in the shoots after 6 h of NaCl treatment, as seen in Supplementary Table S9. Despite the finding that fewer *POD* genes were upregulated than downregulated, one *POD* (*POD43*) was not expressed under the control condition, whereas its expression was substantially induced in the shoots after 6 h of NaCl treatment, as seen in Figure 4C and Table S9. Almost all the differentially expressed peroxisomal biogenesis factor-encoding genes (*PEXs*), *Trxs*, and peroxiredoxin-encoding genes (*PrxRs*) in shoots after 6 h of salt treatment were upregulated (including 1 *PEX* and 2 *Trx*s, showing no expression under the control condition but high expression under salt treatment), as seen in Figure 4C and Table S9, indicating that the CAT and PrxR/Trx pathways mainly function in the shoot ROS-scavenging system of *P. cornutum* under salt treatment. Following 24 h of salt treatment, only a few genes associated with ROS scavenging were differentially expressed in shoots, and most of these genes were downregulated, as seen in Figure 4D. However, *PEX3-1a* showed no expression under the control condition, but showed upregulated expression after both 6 and 24 h of salt treatment, as seen in Supplementary Tables S9 and S10, suggesting that *PEX3-1a* might play an essential role in ROS scavenging in shoots of *P. cornutum* under saline conditions.

### 3.6. DEGs Related to Photosynthesis

The physiological analysis suggested that the maintenance of high carbon assimilation efficiency might be another important trait for adaptation of *P. cornutum* to 50 mM NaCl, as seen in Figure 1. The oxygenic photosynthesis of higher plants consists of a photosynthetic electron transport system with many components, such as chlorophyll, photosystem II-light harvesting complex (PS II), photosystem I-light harvesting complex (PS I), cytochrome *b_6_f* complex, ferredoxin, ATP synthase, and various carbon-fixing enzymes [25]. Thus, we analyzed the expression of DEGs related to the abovementioned components in *P. cornutum* treated with 50 mM NaCl.

**Figure 5:**
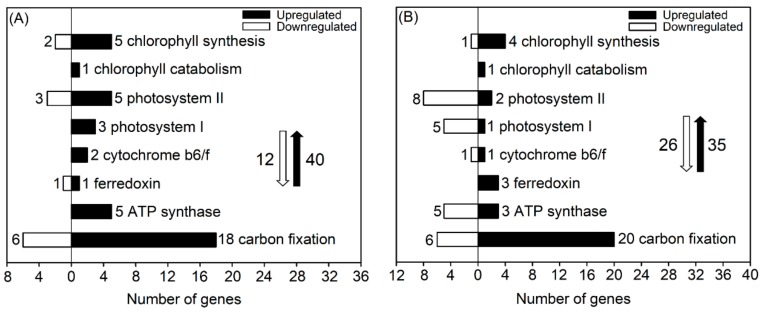
Number of DEGs related to photosynthesis in shoots of *P. cornutum* under 50 mM NaCl treatment for 6 h (**A**) and 24 h (**B**). The white downward and black upward arrows show the total number of downregulated DEGs and upregulated DEGs, respectively.

Under 50 mM NaCl treatment for 6 h, a majority of DEGs related to the components of the PS II complex and chlorophyll biosynthesis and all of the DEGs related to the components of the PS I complex, cytochrome *b*_6_*f* complex, and ATP synthase in shoots of *P. cornutum* were upregulated, as seen in Figure 5A, suggesting that the expression of these genes in shoots might be essential for light energy absorption and photosynthetic electron transport to generate more ATP for carbon fixation. Furthermore, nearly 20 DEGs encoding various enzymes (mainly malate dehydrogenase and phosphoglycerate kinase) in the carbon fixation process were upregulated, as seen in Figure 5A and Supplementary Table S11, indicating a vital role for these genes in the maintenance of high carbon assimilation efficiency in *P. cornutum* under salt stress.

Under 50 mM NaCl treatment for 24 h, the numbers of upregulated DEGs in shoots related to the components of the PS II complex, PS I complex, chlorophyll biosynthesis, and ATP synthase were much lower than those under NaCl treatment for 6 h, but the expression of one cytochrome f-encoding gene, one ferredoxin-dependent glutamate synthase-encoding gene, and one uroporphyrinogen III methyltransferase-encoding gene was still upregulated, as seen in Figure 5B and Supplementary Table S12. These three genes might play a crucial role in light energy absorption and photosynthetic electron transport in *P. cornutum* under saline conditions. Furthermore, a majority of the upregulated DEGs involved in carbon fixation after 6 h of salt treatment were also upregulated after 24 h of salt treatment, as seen in Figure 5 and Tables S11 and S12.

### 3.7. DEGs Related to Transcription Factors

Along with functional genes, regulatory genes also participate in the responses of plants to environmental stress by regulating signal transduction or functional gene expression [26]. Transcription factors (TFs) are important and abundant regulatory genes in higher plants. The major classes of TFs include no apical meristem/Arabidopsis transcription activation factor/cup shaped cotyledon (NAC), APETALA2 and ethylene-responsive element binding proteins (AP2/ERF), basic helix-loop-helix (bHLH), myeloblastosis (MYB), WRKY-domain protein (WRKY), basic region-leucine zipper/homeodomain-leucine zipper (bZIP/HD-ZIP), zinc finger (ZF), and heat shock protein (HSP), some of which have been shown to confer salt and drought tolerance in various plant species by transcriptional regulation of target downstream stress-responsive genes [26]. As *TFs* generally show rapid responses to abiotic stress, we only analyzed the differentially expressed TF genes in roots and shoots after 6 h of salt treatment (the number of differentially expressed TF genes after salt treatment for 24 h was much lower than that after salt treatment for 6 h in our results; data not shown).

**Figure 6:**
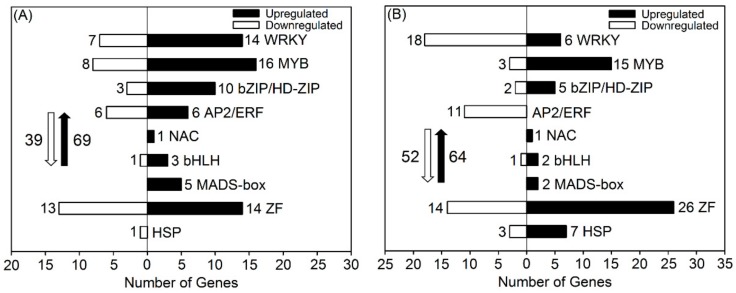
Number of DEGs related to transcription factors in roots (**A**) and shoots (**B**) of *P. cornutum* under 50 mM NaCl treatment for 6 h. The white downward and black upward arrows show the total number of downregulated DEGs and upregulated DEGs, respectively.

After 6 h of mM NaCl treatment, more than 100 TF genes were differentially expressed in roots; approximately two thirds were upregulated, a majority of which were *WRKYs*, *MYBs*, *ZFs*, or *bZIPs*/*HD-ZIPs,* including *WRKY33*, *WRKY54*, *MYB3*, CCCH-type *ZFs*, and *bZIP24*, as seen in Figure 6A and Supplementary Table S13. Although little research is available showing that *MADS-box* genes are closely related to the responses of plants to salinity, all five differentially expressed *MADS-box* genes in roots after salt treatment were considerably upregulated, with three members (*AGL16*, *AGL27*, and *AGL29*) showing no expression under the control condition, but high expression under salt treatment, as seen in Figure 6A and Table S13. Up to six upregulated AP2/ERF-encoding genes were also found in roots. Only a few NAC, bHLH, and HSP-encoding genes were differentially expressed after salt treatment, as seen in Figure 6A.

After 6 h of 50 mM NaCl treatment, nearly 120 TF genes were differentially expressed in shoots, as seen in Figure 6B. The number of upregulated WRKY- and AP2/ERF-encoding genes in shoots was much lower than that in roots, as seen in Figure 6, suggesting that *WRKYs* and *AP2/ERFs* might mainly regulate stress-responsive functional genes in roots to confer salt tolerance in *P. cornutum*. Similarly, the upregulated MADS-box-encoding genes were less abundant in shoots than in roots after salt treatment, but the expression of *AGL30* was upregulated more than five-fold in both shoots and roots, as seen in Figure 6 and Tables S13 and S14. Many upregulated *MYBs* and *ZFs* (six and seven members, respectively) that showed no expression under the control condition, were also observed in shoots after salt treatment, as seen in Figure 6B and Table S14. Notably, the upregulated HSP-encoding genes were found in shoots but not in roots, as seen in Figure 6, suggesting that *HSPs* might mainly regulate stress-responsive functional genes in shoots of *P. cornutum* under saline conditions.

### 3.8. Validation of the RNA-seq Results

**Figure 7:**
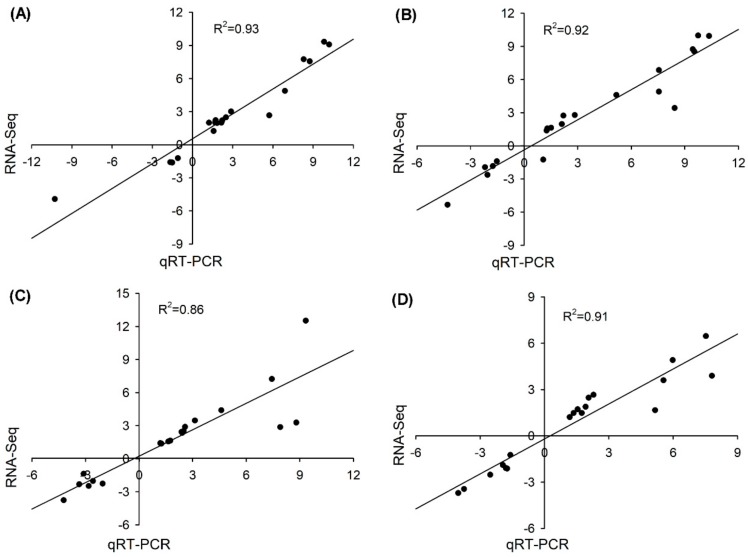
Correlation analysis for expression pattern validation of 20 randomly selected DEGs under 50 mM NaCl for 6 h and 24 h in roots ((**A**,**B**), respectively) and in shoots ((**C**,**D**), respectively) by qRT-PCR. The X-axes and Y-axes show the gene transcript level changes obtained by qRT-PCR and RNA-seq, respectively. R^2^ indicates the correlation.

The transcript abundance of 40 randomly selected DEGs (20 from roots and 20 from shoots) was determined using quantitative real-time PCR (qRT-PCR) to validate the RNA-seq data. As shown in Supplementary Tables S15 and S16, the fold changes of the selected DEGs under salt treatment measured by qRT-PCR were highly consistent with the results obtained from the RNA-seq data. Moreover, the correlation coefficient (R^2^) of the DEGs from roots between the qRT-PCR and RNA-seq results under salt treatment for 6 and 24 h was 0.93 and 0.92, respectively, as seen in Figure 7A,B, and the R^2^ of the DEGs from shoots between the qRT-PCR and RNA-seq results under salt treatment for 6 and 24 h was 0.86 and 0.91, respectively, as seen in Figure 7C,D, indicating that the RNA-seq data in the present study were highly reliable.

## 4. Discussion

### 4.1. The Possible Molecular Basis Underlying Cl^−^ Transport in the Cl^−^-Accumulating Species P. cornutum 

For most crops, especially legumes and perennial woody species, Cl^−^ toxicity is a major cause of yield reduction under saline conditions [13]. However, it has been proven that the considerable absorption of Cl^−^ from external surroundings and translocation of Cl^−^ from roots into shoots for osmotic adjustment are important physiological mechanisms underlying the salt tolerance of the xerophyte *P. cornutum,* which can adapt well to halomorphic arid soils [11,12]. Therefore, *P. cornutum* must have evolved multiple molecular mechanisms for Cl^−^ transport to survive in these harsh conditions.

As a micronutrient, Cl^−^ is primarily absorbed by the epidermal cells of root hairs via several transporters and channels [27]. Decades ago, active Cl^−^ transporters such as Cl^−^/2H^+^ symporters and passive Cl^−^ influx/efflux channels in the plasma membrane of the root epidermis were identified using electrophysiological approaches [28], but to date, the corresponding candidate genes remain unknown. A previous study on *Zea mays* proposed that ZmNPF6.4 is likely a component of the plant root Cl^−^ uptake system, as ZmNPF6.4 is a plasma membrane-localized, proton-coupled, chloride-selective transporter in root epidermal cells [29]. However, the transcriptional responses of *NPF6.4* in plants to salt stress have not been investigated. In the present study, two transcripts of *NPF6.4*, namely, *NPF6.4a* (CL7387. Contig1_All) and *NPF6.4b* (CL7387. Contig2_All), were found in *P. cornutum*, and the transcript abundance of *NPF6.4a* in roots was continuously induced by 50 mM NaCl treatment for both 6 and 24 h, as seen in Table 2 and Tables S3 and S4, indicating that *NPF6.4a* might be a candidate protein that facilitates the uptake of Cl^−^ by roots in *P. cornutum* under saline conditions.

Overaccumulation of Cl^−^ in shoots generally triggers Cl^−^ toxicity in photosynthetic organs and ultimately restricts plant growth [27]. The key rate-limiting gatekeeper step modulating Cl^−^ accumulation in the shoots has been shown to be the loading of Cl^−^ from the root stelar symplast into the xylem apoplast [13]. SLAH1 is currently the only known protein that mediates Cl^−^ loading into the xylem under saline conditions [30]. In the Cl^−^-sensitive plant *A. thaliana*, the expression of *AtSLAH1* in roots is substantially downregulated under salt stress [30], indicating that Cl^−^-sensitive species mainly avoid Cl^−^ overaccumulation by repressing the expression of *SLAH1* in roots. Conversely, in the roots of *P. cornutum*, *SLAH1* showed no expression under normal conditions but was highly expressed under salt treatment for both 6 and 24 h, as seen in Table 2 and Tables S3 and S4, indicating that *P. cornutum* likely accelerates its Cl^−^ accumulation by increasing the expression of *SLAH1* in roots. Therefore, the function of *SLAH1* in the regulation of salt tolerance should be distinct between salt-sensitive and salt-tolerant plant species. CCC1 may be also involved in the loading of Cl^−^ into the root xylem [31]. Our results showed that the transcript abundance of *CCC1* in roots of *P. cornutum* was downregulated under salt treatment, as seen in Figure 3B, which might be related to the change of plant root pressure, as suggested by Wegner 2014 [32].

AtCLCg is a unique protein that mediates Cl^−^ compartmentalization into vacuoles of mesophyll cells in Arabidopsis [33]. In the typical Cl^−^-sensitive plant *Glycine max*, *GmCLC1*, a homolog of *AtCLCg* is preferentially expressed at the tonoplast of root cells, and thus the main function of GmCLC1 is to withhold Cl^−^ in roots to decrease Cl^−^ accumulation in shoots [34]. In the present study, the transcript abundance of *P. cornutum CLCg* in shoots was enhanced under salt treatment at both 6 and 24 h, as seen in Table 3. Considering that *P. cornutum* is a typical Cl^−^-accumulating plant, the expression of *CLCg* in shoots under salt treatment might be essential for Cl^−^ accumulation in the vacuoles of the succulent shoot tissue to enhance osmotic adjustment capacity. Therefore, there should be distinctly different models for the response of *CLCg* to salt stress between Cl^−^-sensitive and Cl^−^-tolerant species.

Taken together, the integrated response of genes associated with Cl^−^ transport, especially *NPF6.4*, *SLAH1*, and *CLCg*, should be of particular importance for conferring pronounced salt tolerance in *P. cornutum* compared to other plant species.

### 4.2. The Transport of Other Important Ions Plays a Vital Role in the Response to Salt Stress in P. cornutum

The uptake and storage of NO_3_^−^ in plants under saline conditions is generally antagonized by the uptake of Cl^−^, due to the competition between Cl^−^ and NO_3_^−^ for the major binding sites of transmembrane channels or transporters [13]. Our previous studies have shown that the capacity to maintain NO_3_^−^ homeostasis in shoots is also a principal trait associated with the Cl^−^ accumulation capability of *P. cornutum* under saline conditions [11,12]. The nitrate transporter NPF6.3 (also known as CHL1 or NRT1.1) has been identified as a key protein that mediates NO_3_^−^ uptake by plant roots [35]. Our results identified two transcripts for *NPF6.3*, namely, *NPF6.3a* (CL8124. Contig1_All) and *NPF6.3b* (CL8124. Contig3_All), in *P. cornutum*; the former was upregulated after 24 h of salt treatment in the roots, and the latter was upregulated after 6 h of salt treatment in the roots, as seen in Tables S3 and S4. These two NPF6.3 proteins might play an essential role in salt tolerance in *P. cornutum* by mediating NO_3_^−^ uptake in roots. NPF7.3 (also known as NRT1.5) was shown to participate in the root-to-shoot transport of NO_3_^−^ in plants [36]. In *A. thaliana*, *AtNPF7.3* expression in roots is downregulated in response to NaCl treatment [37]. However, the expression of *NPF7.3* in *P. cornutum* remained unchanged under salt treatment (because *NPF7.3* was not found among the DEGs) to render constant NO_3_^−^ transport from roots into shoots under salt treatment. Therefore, the superior long-distance transport ability of NO_3_^−^ regulated by *NPF7.3* likely represents another important trait associated with the salt tolerance of *P. cornutum*.

Efficient maintenance of Na^+^ and K^+^ homeostasis is crucial for salt tolerance in higher plants [1]. Numerous proteins involved in these processes have been identified and functionally characterized. For example, CNGC, GLR, and NCX are involved in root Na^+^ and/or K^+^ uptake [38,39,40], HKT1 and SOS1 mediate long-distance Na^+^ transport from roots to shoots [41,42], KT/KUP/HAK mediate K^+^ transport in roots [43], and tonoplast NHX mediates Na^+^ and/or K^+^ compartmentalization into vacuoles [44]. In the present study, we found that many genes encoding the abovementioned proteins were upregulated after salt treatment, as seen in Figure 3, indicating that *P. cornutum* is able to maintain cationic homeostasis under saline conditions by increasing the expression of these genes. It has been proven that *Zygophyllum xanthoxylum*, another important xerophyte in northwestern China, can accumulate extremely high concentrations of Na^+^ as an important osmoticum in leaves to improve photosynthesis and hydration under salt and drought stress [16]. Subsequent studies have validated the vital roles of some Na^+^ and/or K^+^ transporters/channels in the maintenance of Na^+^/K^+^ homeostasis in *Z. xanthoxylum* [45,46]. Therefore, the molecular mechanisms underlying Na^+^ and K^+^ transport in xerophytic desert plants are much clearer than those involved in Cl^−^ transport. However, our results also provide new insights into Na^+^/K^+^ homeostasis in desert plants. For example, the transcript abundance of *HKT1* in *P. cornutum* was upregulated in shoots but remained stable in roots under salt treatment for both 6 and 24 h, as seen in Figure 3 and Table 3, indicating that *HKT1* is mainly responsive to salt in shoots of *P. cornutum*. To date, the molecular factors involved in Na^+^ transport into mesophyll cells in plants remain unknown. As HKT1-type proteins are located at the plasma membrane and mediate Na^+^ transport [41], the expression of *HKT1* in shoots might contribute to the entry of Na^+^ into shoots for using Na^+^ as an osmoticum in *P. cornutum*. Furthermore, the transcript abundances of *NHX5* and *NHX6* were upregulated in the roots and shoots of *P. cornutum* under salt treatment for both 6 and 24 h, as seen in Table 2; Table 3. In *A. thaliana*, both NHX5 and NHX6 are Golgi and trans-Golgi network-located Na^+^/H^+^ antiporters that synergistically trigger plant salt tolerance by maintaining organelle pH and ion homeostasis [47]. However, the function of *NHX5* and *NHX6* in the salt tolerance of desert plants has not been investigated. Our results suggested that both *NHX5* and *NHX6* might also be important candidates for the adaptation of desert plants to harsh environments.

### 4.3. The ROS-Scavenging System is Important for Salt Stress Adaptation in P. cornutum

SOD is the primary antioxidant enzyme in the ROS-scavenging system. It can convert superoxide anion radicals into free oxygen radicals and hydrogen peroxide. Subsequently, large amounts of the resulting free oxygen radicals and hydrogen peroxide are scavenged mainly through the ASA–GSH cycle, the GPX pathway, the CAT pathway, and the PrxR/Trx pathway [48]. SODs in higher plants are classified by their metal cofactors into three known types, namely, Cu/Zn SOD, Mn SOD and Fe SOD, among which Cu/Zn SOD has the widest subcellular distribution (cytosolic fraction and chloroplast) [49]. In this study, the transcript abundance of *Cu-Zn SOD* was upregulated after salt treatment for 24 h in both roots and shoots of *P. cornutum*, as seen in Figure 4 and Tables S8 and S10. Hence, Cu-Zn SOD should be a core component in the ROS-scavenging system of *P. cornutum* confronted with salinity. CAT is indispensable for ROS detoxification during stress conditions; it has the highest efficiency for converting H_2_O_2_ in plant cells into H_2_O and O_2_ among all the antioxidant enzymes in plants [48]. In the present study, only *CAT3* was induced in roots and shoots by salt treatment, as seen in Figure 4 and Tables S8 and S10, suggesting that under saline conditions, *P. cornutum* mainly increases CAT activity by upregulating the expression of *CAT3* in roots and shoots. Among the upregulated DEGs involved in the four ROS-scavenging pathways in roots and shoots after salt treatment, most were *GSTs* and *Trxs*, as seen in Figure 4. In the AsA–GSH cycle and GPX pathway, GSH acts as a redox sensor to maintain relatively low levels of ROS; it must be first catalyzed by GSTs as an antioxidant, after which it can function in ROS scavenging [48]. Many studies have found that overexpression of *GSTs* could substantially improve ROS-scavenging capacity in plants under salt stress [48]. Therefore, the upregulated *GSTs* identified in the current study probably also play vital roles in the ROS-scavenging system of *P. cornutum* under saline conditions, as seen in Figure 4. The thioredoxin (Trx) proteins serve as redox transmitters within the cellular thiol/disulfide redox network, and the peroxiredoxin (PrxR) proteins act as thiol-dependent peroxidases with high affinity for peroxides, especially for H_2_O_2_, to protect protein thiols from oxidation. Thus, the PrxR/Trx pathways also function in the ROS-scavenging system in plants [50]. In the present study, many *Trx* genes in roots and shoots were upregulated after salt treatment, as seen in Figure 4, suggesting that Trx may play essential roles in the ROS-scavenging system of *P. cornutum* under NaCl treatment.

### 4.4. High Efficiency for Stomatal Movement and Carbon Fixation is Essential for the Adaptation of P. Cornutum to Salt Stress

Stomatal closure to reduce water loss through transpiration is an important strategy adopted by *P. cornutum* to adapt to salt stress, which in turn triggers stomatal limitation for photosynthesis; however, although the net photosynthetic rate of *P. cornutum* is significantly decreased, its dry biomass remained stable due to its high carbon assimilation capacity, as seen in Figure 1. Therefore, the genes implicated in stomatal movement and carbon assimilation should play essential roles in salt tolerance in *P. cornutum*.

The aperture of the stomatal pore is regulated by changes in the osmotic potentials of the guard cells. These changes are mainly achieved by transport of K^+^ and organic as well as inorganic anions across cellular membranes [51]. The outward K^+^ channel GORK participates in K^+^ efflux through the plasma membrane of guard cells and is consequently involved in stomatal movement [52]. In the present study, salt-induced expression of *GORK* in the shoots of *P. cornutum* was observed, as seen in Figure 3D, suggesting that this gene is closely related to stomatal closure in *P. cornutum* confronted with salt stress. In *A. thaliana*, several anion transporters also mediate NO_3_^−^ and/or Cl^−^ transport across the plasma membrane or tonoplast of guard cells. For example, the slowly activated anion conductance 1 (SLAC1) and aluminum-activated malate transporter 12 (ALMT12) mediate NO_3_^−^ and/or Cl^−^ release or uptake from guard cells, and CLCc and ALMT9 mediate Cl^−^ compartmentalization into vacuoles of guard cells [53,54,55]. In *P. cornutum*, the transcript abundance of these genes in shoots was unaltered after salt treatment, indicating that there should be other components involved in anion release or uptake from guard cells.

Photosynthetic electron transport is the primary step in the process of oxygenic photosynthesis, which converts sunlight into active chemical energy to provide ATP for the subsequent carbon assimilation [25]. In the present study, numerous upregulated genes associated with all the major components of photosynthetic electron transport, such as chlorophyll biosynthesis, the PS II complex, the PS I complex, the cytochrome *b_6_f* complex, ATP synthase and ferredoxin, were identified in the shoots of *P. cornutum* after salt treatment for either 6 or 24 h, as seen in Figure 5. These photosynthetic electron transport-related genes in the shoots of *P. cornutum* are probably involved in the enhancement of photosynthetic performance by strong activation of light energy conversion and ATP generation. Furthermore, our results identified many upregulated genes that encode various enzymes directly involved in carbon assimilation, including genes encoding phosphoenolpyruvate carboxylase, malate dehydrogenase, alanine aminotransferase/transaminase, ribulose-1,5-diphosphate carboxylase, phosphoglycerate kinase, and glyceraldehyde-3-phosphate dehydrogenase-encoding genes, as seen in Figure 4 and Tables S11 and S12. Hence, these genes likely play central roles in enhancing the carbon assimilation capacity of *P. cornutum* under saline conditions.

### 4.5. Transcription Factors Play Important Roles in Regulating Salt-Responsive Genes in P. Cornutum under Salt Stress

The bZIP TFs play an important and integrative function in salt- and drought-signaling networks of higher plants, especially bZIP24, which has been identified as a key regulator in the salt tolerance of *A. thaliana* because it can stimulate the transcription of a wide range of stress-inducible functional genes such as *HKT1* and *SOS1* [56]. In the present study, the transcript level of *bZIP24* in roots of *P. cornutum* was upregulated after salt treatment, as seen in Table S13, suggesting that *bZIP24* in roots might predominately regulate transcriptional networks in salt stress adaptation of *P. cornutum*. In addition, many other salt-inducible *HD-ZIPs*, which have rarely been reported in plants, were also identified in the roots and shoots of *P. cornutum*, as seen in Supplementary Tables S13 and S14. These *HD-ZIPs* might play specific roles in modulating the salt tolerance of *P. cornutum*. WRKY proteins regulate diverse plant processes, including biotic and abiotic stress adaptation. In *A. thaliana*, WRKY33 is indispensable for salt tolerance [57]. In our study, one *WRKY33* transcript, *WRKY33a*, was highly induced by salt in both roots and shoots of *P. cornutum*, and another transcript, *WRKY33b*, was induced by salt in roots, as seen in Tables S13 and S14. Therefore, WRKY33 likely also plays essential roles in salt signaling networks in *P. cornutum*. The Cys2/His2-type ZF proteins have been proven to control and regulate WRKY functions, the ROS-signaling pathway and stomatal closure [58]. In our results, two transcripts (CL7040. Contig1_All and CL7040. Contig2_All) for *Cys2/His2-2* were both induced by salt treatment in shoots, as seen in Table S14, indicating that these two genes might also be important for salt tolerance in *P. cornutum*. In addition to Cys2/His2-type ZF proteins, certain members of the bHLH family, such as bHLH92, confer salt tolerance in plants by controlling ROS-scavenging-related signaling, as various PODs are the downstream targets [59]. In *P. cornutum*, the transcript abundance of *bHLH92* in shoots was upregulated after salt treatment, as seen in Table S14. Thus, *bHLH92* likely participates in the enhancement of the ROS-scavenging capacity in the salt adaptation of *P. cornutum.* Additionally, our results identified some other salt-inducible TF genes in *P. cornutum,* such as *NAC62* and *MYB59*, as seen in Tables S13 and S14, the regulatory mechanisms of which in salt adaptation in other plants have been less intensively investigated. These genes might represent novel regulators involved in salt tolerance in *P. cornutum*.

## 5. Conclusions

This study provides the first analysis of gene transcripts in *P. cornutum* under salt treatment. Candidate genes that most likely confer salt tolerance to *P. cornutum* by facilitating Cl^−^ accumulation and NO_3_^−^ homeostasis, as well as the transport of other inorganic osmoticums, such as Na^+^ and K^+^, were identified. Meanwhile, many salt-responsive genes associated with the enhancement of ROS-scavenging capacity and carbon assimilation efficiency to improve the salt tolerance of *P. cornutum* were identified. Additionally, possible transcription factor genes with multiple functions in the regulation of the adaptation of *P. cornutum* to salt stress were found. These results promote research on the molecular mechanisms of salt tolerance in xerophytic species and lay a foundation for genetic improvement of the stress resistance of important forage and crop species in arid areas.

## Figures and Tables

**Table 1:** Summary of de novo sequence assembly.

Unigenes	Total Number	Total Length (bp)	Mean Length (bp)
Shoots	64,978	70,865,211	1091
Roots	80,307	80,091,654	997
All	72,086	89,575,317	1243

**Table 2:** Upregulated DEGs related to ion transport in roots of *P. cornutum* after 50 mM NaCl treatment for both 6 and 24 h.

Gene ID	Gene Name	Function
CL5647.Contig1_All	*SLAH1*	Cl^−^ transport from roots to shoots
CL2477.Contig4_All	*CLCg*	Cl^−^ vacuole compartmentalization
CL7387.Contig1_All	*NPF6.4*	Cl^−^ uptake at root epidermis
CL1096.Contig4_All	*NHX5*	Na^+^/H^+^ antiporter
CL1096.Contig10_All	*NHX6*	Na^+^/H^+^ antiporter
CL3604.Contig8_All	*GLR3.3*	Cation channel activator
CL3206.Contig3_All	*Sultr1.2*	SO_4_^2−^ uptake
CL7785.Contig1_All	*Sultr3.3*	SO_4_^2−^ transporter
CL564.Contig8_All	*PHT4.3*	PO_4_^3−^ transporter
CL2740.Contig1_All	*PDR2*	Mn^2+^ transporter
CL666.Contig1_All	*BOR4*	BO_3_^3−^ exporting from cytoplasm

**Table 3:** Upregulated DEGs related to ion transport in shoots of *P. cornutum* after 50 mM NaCl treatment for both 6 and 24 h.

Gene ID	Gene Name	Function
CL4002.Contig2_All	*CCC1*	Cl^−^ retrieval from xylem sap
CL2577.Contig2_All	*CLCf*	Cl^−^ channel
CL2477.Contig4_All	*CLCg*	Cl^−^ vacuole compartmentalization
Unigene7084_All	*NPF5.2*	Cl^−^ and/or NO_3_^−^ transporter
CL3282.Contig2_All	*NPF6.2*	Cl^−^ and/or NO_3_^−^ transporter
CL6278.Contig1_All	*NPF8.1*	Cl^−^ and/or NO_3_^−^ transporter
CL2487.Contig3_All	*NPF5.9*	Cl^−^ and/or NO_3_^−^ transporter
CL1096.Contig4_All	*NHX5*	Na^+^/H^+^ antiporter
CL1096.Contig10_All	*NHX6*	Na^+^/H^+^ antiporter
CL3834.Contig2_All	*HKT1*	Na^+^ and/or K^+^ cellular efflux
CL1280.Contig5_All	*KUP5*	K^+^ transporter
Unigene6900_All	*CNGC1*	Ca^2+^ root uptake
Unigene13264_All	*CTR5*	Cu^2+^ transporter
Unigene1617_All	*MGT10*	Mg^2+^ transporter

## Supplementary Materials

The following are available online at https://www.mdpi.com/2073-4425/10/12/1039/s1, Figure S1: Length distribution of all assembled unigenes. Table S1: Sequencing production statistics. Table S2: Summary of sequence annotation. Table S3: Differentially expressed genes (DEGs) related to ion transport in roots of *P. cornutum* after 50 mM NaCl treatment for 6 h. Table S4: Differentially expressed genes (DEGs) related to ion transport in roots of *P. cornutum* after 50 mM NaCl treatment for 24 h. Table S5: Differentially expressed genes (DEGs) related to ion transport in shoots of *P. cornutum* after 50 mM NaCl treatment for 6 h. Table S6: Differentially expressed genes (DEGs) related to ion transport in shoots of *P. cornutum* after 50 mM NaCl treatment for 24 h. Table S7: Differentially expressed genes (DEGs) related to ROS-scavenging system in roots of *P. cornutum* after 50 mM NaCl treatment for 6 h. Table S8: Differentially expressed genes (DEGs) related to ROS-scavenging system in roots of *P. cornutum* after 50 mM NaCl treatment for 24 h. Table S9: Differentially expressed genes (DEGs) related to ROS-scavenging system in shoots of *P. cornutum* after 50 mM NaCl treatment for 6 h. Table S10: Differentially expressed genes (DEGs) related to ROS-scavenging system in shoots of *P. cornutum* after 50 mM NaCl treatment for 24 h. Table S11: Differentially expressed genes (DEGs) related to photosynthesis in shoots of *P. cornutum* after 50 mM NaCl treatment for 6 h. Table S12: Differentially expressed genes (DEGs) related to photosynthesis in shoots of *P. cornutum* after 50 mM NaCl treatment for 24 h. Table S13: Differentially expressed genes (DEGs) related to transcript factors in roots of *P. cornutum* after 50 mM NaCl treatment for 6 h. Table S14: Differentially expressed genes (DEGs) related to transcript factors in shoots of *P. cornutum* after 50 mM NaCl treatment for 6 h. Table S15: Expression pattern validation of 20 randomly selected genes in roots of *P. cornutum* under 50 mM NaCl treatment for 6 and 24 h by qRT-PCR method. Table S16: Expression pattern validation of 20 randomly selected genes in shoots of *P. cornutum* under 50 mM NaCl treatment for 6 and 24 h by qRT-PCR method.
