# Supplementary material for: Transcriptomic Profiling Identifies Candidate Genes Involved in the Salt Tolerance of the Xerophyte Pugionium cornutum
Source: Genes (Basel). 2019 Dec 12;10(12):1039. doi: 10.3390/genes10121039 (PMC6947847; doi:10.3390/genes10121039)
Supplement: Supplementary file 1 [file genes-10-01039-s001.pdf]

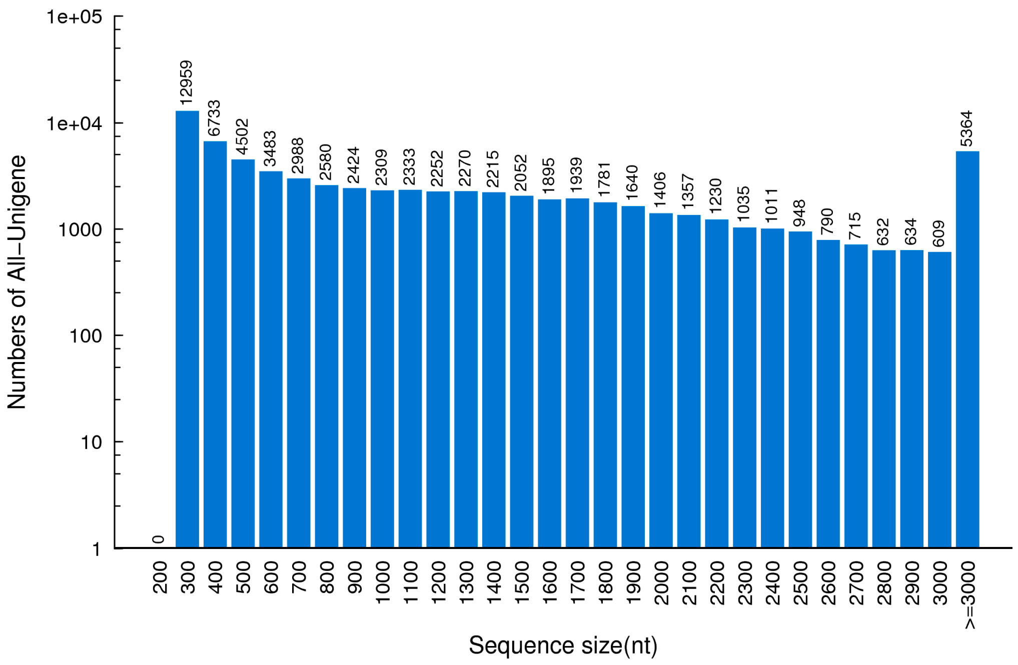

Figure S1. Length distribution of all assembled unigenes.

Table S1. Sequencing production statistics.

Samples	Total Raw Reads	Total Clean Reads	GC percentage
Shoot	118059830	115777772	45.01%
Root	119410188	117031190	46.26%

Table S2. Summary of sequence annotation.

Database	NR	NT	Swiss-Prot	KEGG	COG	GO	All
Numbers	57695	60628	39893	35136	24608	53435	63396
Percentage	80.01%	84.11%	55.34%	48.74%	34.14%	74.13%	87.94%

Table S3. Differentially expressed genes (DEGs) related to ion transport in roots of *P. cornutum* after 50 mM NaCl treatment for 6 h. C6R RPKM and S6R RPKM indicates the RPKM value of a gene in roots under control condition for 6 h and salt treatment for 6 h respectively. Fold change equals to $\log_2$(S6R RPKM / C6R RPKM) and indicates the transcript abundance change of each DEGs. Protein is the protein encoded by each DEGs. The Gene ID of each upregulated DEGs with no expression under control condition is in bold.

Gene ID	C6R RPKM	S6R RPKM	Log$_2$ Ratio	Protein	Most homologous species
**CL5647.Contig1_All**	0.01	3.42	8.42	SLAH1	*Arabidopsis thaliana*
**CL4502.Contig2_All**	0.01	6.64	9.38	CLCa	*Arabidopsis thaliana*
CL2477.Contig4_All	3.02	6.3	1.06	CLCg	*Camelina sativa*
Unigene6878_All	0.19	1.72	3.18	NPF5.1	*Camelina sativa*
CL8124.Contig3_All	4.14	26.37	2.67	NPF6.3b	*Eutrema salsugineum*
Unigene20640_All	1.18	6.29	2.41	NPF2.6	*Eutrema salsugineum*
Unigene16336_All	5.37	10.66	1.99	NPF8.1b	*Brassica napus*
CL1892.Contig3_All	7.52	18.06	1.26	NPF2.11	*Eutrema salsugineum*
CL7387.Contig1_All	0.04	0.09	1.17	NPF6.4a	*Eutrema salsugineum*
Unigene17986_All	0.52	1.13	1.12	NPF5.4	*Brassica napus*
CL3282.Contig3_All	10.49	5.24	-1.01	NPF6.2	*Eutrema salsugineum*

Unigene2805_All	3.66	1.7	-1.11	NPF3.1	*Eutrema salsugineum*
CL1188.Contig3_All	1.36	0.01	-7.09	NPF8.4	*Eutrema salsugineum*
**CL141.Contig3_All**	0.01	0.44	5.46	SOS1	*Brassica rapa*
**CL1096.Contig10_All**	0.01	1.39	7.12	NHX6	*Arabidopsis thaliana*
CL1096.Contig4_All	0.63	2.27	1.85	NHX5	*Arabidopsis thaliana*
CL141.Contig5_All	1.56	0.01	-7.29	NHX8	*Camelina sativa*
CL6534.Contig4_All	21.32	10.25	-1.06	NCX	*Arabidopsis thaliana*
**CL3604.Contig8_All**	0.01	4.61	8.85	GLR3.3a	*Brassica napus*
**CL415.Contig2_All**	0.01	0.79	6.30	GLR3.2	*Arabidopsis thaliana*
CL5397.Contig2_All	0.78	8.44	3.44	GLR2.5	*Camelina sativa*
CL3604.Contig12_All	0.84	3.79	2.17	GLR3.3b	*Camelina sativa*
CL1280.Contig5_All	0.92	5.85	2.67	KUP2	*Arabidopsis thaliana*
Unigene20531_All	8.32	22	1.40	KT3	*Brassica napus*
CL2337.Contig11_All	11.52	5.51	-1.06	KUP10	*Arabidopsis thaliana*
CL9487.Contig2_All	23.25	10.77	-1.11	HAK2	*Arabidopsis thaliana*
Unigene10500_All	28.72	10.46	-1.46	HAK10	*Arabidopsis thaliana*
CL2337.Contig2_All	1.65	0.58	-1.51	HAK9	*Arabidopsis thaliana*
Unigene17269_All	2.66	0.01	-8.06	KT2d	*Camelina sativa*
**CL1585.Contig2_All**	0.01	0.77	6.27	KEA6	*Brassica rapa*
CL6926.Contig3_All	1.73	3.86	1.16	KEA1	*Brassica napus*
CL3137.Contig2_All	7.53	0.01	-9.56	KEA5	*Arabidopsis thaliana*
**CL3663.Contig5_All**	0.01	0.56	5.81	CNGC6a	*Camelina sativa*
Unigene11269_All	6.4	14.37	1.17	CNGC6b	*Arabidopsis thaliana*
CL3663.Contig3_All	2.13	4.62	1.12	CNGC9	*Camelina sativa*
Unigene30942_All	19.84	4.09	-2.28	CNGC6d	*Arabidopsis thaliana*
Unigene17269_All	2.66	0.01	-8.06	CNGC6c	*Arabidopsis thaliana*
**CL3206.Contig3_All**	0.01	1.57	7.29	Sultr1.2a	*Brassica rapa*
Unigene16699_All	0.27	2.76	3.35	Sultr1.2b	*Brassica rapa*
Unigene20847_All	1.66	9.74	2.55	Sultr3.5	*Brassica napus*
Unigene20242_All	4.61	11.14	1.27	Sultr1.1	*Brassica napus*
CL7785.Contig1_All	2.29	5.43	1.25	Sultr3.3	*Brassica napus*
CL6298.Contig1_All	7.09	3.38	-1.07	Sultr3.1	*Arabidopsis thaliana*
**CL564.Contig8_All**	0.01	6.23	9.28	PHT4.3	*Arabidopsis thaliana*
**CL5169.Contig1_All**	0.01	1.1	6.78	PHT2.1	*Camelina sativa*
CL7753.Contig1_All	0.7	4.42	2.66	PHT1.3a	*Brassica napus*
Unigene16890_All	4.59	12.17	1.41	PHT1.3b	*Camelina sativa*
Unigene11734_All	3.59	7.65	1.09	PHO1	*Brassica napus*
CL5434.Contig3_All	1.59	14.72	3.21	ZnT8	*Camelina sativa*
CL9009.Contig1_All	4.7	9.58	1.03	ZnT1	*Camelina sativa*
**Unigene4968_All**	0.01	47.35	12.21	CTR5a	*Brassica napus*
CL5383.Contig1_All	6.4	13.95	1.12	CTR2	*Brassica rapa*
CL2038.Contig2_All	2.35	0.01	-7.88	CTR CCHb	*Brassica napus*
CL2038.Contig1_All	1.2	0.01	-6.91	CTR CCHa	*Brassica rapa*
Unigene29796_All	48.01	0.01	-12.23	CTR5c	*Brassica napus*
**CL2740.Contig1_All**	0.01	11.53	10.17	MnT PDR2	*Camelina sativa*
CL5434.Contig1_All	27.17	61.31	1.17	IRT1	*Brassica rapa*
**CL666.Contig5_All**	0.01	2.54	7.99	BOR4b	*Arabidopsis thaliana*
**CL666.Contig2_All**	0.01	0.99	6.63	BOR4a	*Arabidopsis thaliana*
CL6525.Contig1_All	1.87	0.01	-7.55	BOR3	*Arabidopsis thaliana*
CL4850.Contig2_All	0.81	7.42	3.20	V-H^+ ATPase h	*Brassica napus*
Unigene5842_All	18.31	42.37	1.21	V-H^+ ATPase e3	*Arabidopsis thaliana*
Unigene4758_All	15.72	31.65	1.01	V-H^+ ATPase g2	*Camelina sativa*
CL1819.Contig2_All	13.69	98.18	2.84	P-H^+ ATPase	*Arabidopsis thaliana*
Unigene18921_All	1.44	6.8	2.24	P-H^+ ATPase 7a	*Arabidopsis thaliana*
Unigene17708_All	0.99	4.57	2.21	P-H^+ ATPase 7b	*Arabidopsis thaliana*
Unigene19782_All	1.83	6.31	1.79	P-Ca^{2+} ATPase 7	*Brassica rapa*

Table S4. Differentially expressed genes (DEGs) related to ion transport in roots of *P. cornutum* after 50 mM NaCl treatment for 24 h. C24R RPKM and S24R RPKM indicates the RPKM value of a gene in roots under control condition for 24 h and salt treatment for 24 h respectively. Fold change equals to $\log_2$ (S24R RPKM / C24R RPKM) and indicates the transcript abundance change of each DEGs. Protein is the protein encoded by each DEGs. The Gene ID of each upregulated DEGs with no expression under control condition is in bold.

Gene ID	C24R RPKM	S24R RPKM	Log_2 Ratio	Protein	Most homologous species
**CL5647.Contig1_All**	0.01	2.13	7.73	SLAH1	*Arabidopsis thaliana*
Unigene29334_All	7.91	0.59	-3.74	CCC1	*Raphanus Sativus*
**CL2477.Contig4_All**	0.01	6.31	9.30	CLCg	*Camelina sativa*
CL2577.Contig2_All	1.14	9.64	3.08	CLCf	*Camelina sativa*
**CL7387.Contig1_All**	0.01	0.08	3	NPF6.4a	*Eutrema salsugineum*
CL1209.Contig4_All	0.26	1.55	2.58	NPF2.13	*Brassica napus*
CL8124.Contig1_All	15.86	55.97	1.82	NPF6.3a	*Eutrema salsugineum*
CL1188.Contig2_All	1.11	3.82	1.78	NPF8.4	*Eutrema salsugineum*
Unigene2805_All	2.76	8.19	1.57	NPF3.1a	*Eutrema salsugineum*
CL3282.Contig2_All	3.14	9.15	1.54	NPF6.2	*Eutrema salsugineum*
Unigene3586_All	23.67	65.48	1.47	NPF5.7	*Eutrema salsugineum*
CL6278.Contig1_All	5.28	12.92	1.29	NPF8.1a	*Eutrema salsugineum*
CL568.Contig1_All	81.87	200.72	1.29	NPF2.1	*Brassica napus*
Unigene16246_All	326.5	671.44	1.04	NPF3.1b	*Eutrema salsugineum*
CL7387.Contig2_All	0.84	0.38	-1.14	NPF6.4b	*Eutrema salsugineum*
Unigene21409_All	2.4	0.18	-3.74	NPF5.9	*Arabidopsis thaliana*
Unigene17986_All	1.83	0.01	-7.52	NPF5.4	*Brassica napus*
Unigene7084_All	3.39	0.01	-8.41	NPF5.2	*Eutrema salsugineum*
CL141.Contig3_All	0.79	0.01	-6.30	SOS1	*Brassica rapa*
**CL1096.Contig4_All**	0.01	1.52	7.25	NHX5	*Arabidopsis thaliana*
**CL1096.Contig10_All**	0.01	0.67	6.07	NHX6	*Arabidopsis thaliana*
CL4173.Contig3_All	6.05	12.19	1.01	NHX1	*Thellungiella halophila*
**CL3604.Contig8_All**	0.01	1.11	6.79	GLR3.3a	*Brassica napus*
CL8010.Contig2_All	2.18	4.63	1.09	GLR3.6	*Camelina sativa*
CL66.Contig1_All	1.4	2.83	1.02	GLR3.4	*Arabidopsis thaliana*
CL5397.Contig1_All	6.39	1.1	-2.54	GLR2.5	*Camelina sativa*
CL415.Contig2_All	1.52	0.2	-2.93	GLR3.2	*Arabidopsis thaliana*
**CL5926.Contig2_All**	0.01	2.77	8.11	KT2c	*Brassica napus*
CL2362.Contig2_All	3.1	12.56	2.02	HAK3	*Arabidopsis thaliana*
CL2337.Contig2_All	1.85	0.64	-1.53	KUP9	*Arabidopsis thalian*
CL596.Contig4_All	0.87	0.01	-6.44	KUP5	*Arabidopsis thaliana*
CL1280.Contig6_All	1.16	0.01	-6.86	KT2a	*Arabidopsis thaliana*
CL1599.Contig1_All	2.35	6.84	1.54	KEA4	*Camelina sativa*
CL3137.Contig2_All	1.61	4.56	1.50	KEA5	*Arabidopsis thaliana*
CL1585.Contig2_All	1.54	0.23	-2.74	KEA6	*Brassica rapa*
CL3568.Contig2_All	2.64	7.13	1.43	CNGC2	*Arabidopsis thaliana*
Unigene30942_All	6.09	1.82	-1.74	CNGC6d	*Camelina sativa*
Unigene5024_All	120.14	31.83	-1.92	CNGC1a	*Camelina sativa*
CL3663.Contig4_All	0.79	0.01	-6.30	CNGC6a	*Arabidopsis thaliana*
**CL9511.Contig3_All**	0.01	67.18	12.71	V-CHX C2	*Arabidopsis thaliana*
CL3206.Contig3_All	2.14	14.28	2.74	Sultr1.2a	*Brassica rapa*
CL7785.Contig1_All	0.44	2.92	2.73	Sultr3.3	*Camelina sativa*
CL6298.Contig1_All	1	4.96	2.31	Sultr3.1	*Camelina sativa*
Unigene9574_All	27.72	63.02	1.18	Sultr2.1	*Arabidopsis thaliana*
CL7451.Contig2_All	2.7	5.56	1.04	Sultr2.2	*Brassica rapa*
**CL564.Contig8_All**	0.01	0.88	6.46	PHT4.3	*Arabidopsis thaliana*
Unigene1737_All	4.91	9.97	1.02	PHT4.2b	*Arabidopsis thaliana*
CL128.Contig2_All	3.26	0.01	-8.35	PHT2.1	*Camelina sativa*

CL5169.Contig1_All	5.93	0.01	-9.21	PHT4.2a	*Arabidopsis thaliana*
CL9009.Contig4_All	3.71	0.78	-2.25	ZnT12	*Brassica napus*
CL1249.Contig2_All	1.44	0.01	-7.17	ZnT1	*Camelina sativa*
**CL8249.Contig3_All**	0.01	5.07	8.99	CTR PAA1	*Brassica rapa*
CL5383.Contig1_All	30.48	6.43	-2.24	CTR2	*Brassica rapa*
CL2038.Contig2_All	146.3	5.86	-4.64	CTR CCH	*Brassica napus*
CL2740.Contig1_All	9.15	23.65	1.37	MnT PDR2	*Camelina sativa*
Unigene29828_All	16.71	6.32	-1.40	MgT	*Camelina sativa*
Unigene17329_All	13.3	34.56	1.38	AMT1.2	*Arabidopsis thaliana*
CL6691.Contig2_All	33.15	71.56	1.11	AMT1.1	*Camelina sativa*
CL1590.Contig1_All	1.74	0.12	-3.86	AMT2	*Brassica rapa*
CL5434.Contig1_All	57.15	21.97	-1.38	IRT1	*Brassica rapa*
CL666.Contig1_All	5.62	16.45	1.55	BOR4a	*Arabidopsis thaliana*
CL3720.Contig3_All	2.9	0.01	-8.18	BOR4b	*Arabidopsis thaliana*
Unigene21511_All	23.88	66	1.47	V-H^+ ATPase b1	*Arabidopsis thaliana*
Unigene606_All	14.35	39.47	1.46	V-H^+ ATPase c1	*Camelina sativa*
Unigene3164_All	36.62	89.93	1.30	V-H^+ ATPase f	*Camelina sativa*
CL6281.Contig4_All	51.85	109.09	1.07	V-H^+ ATPase e2	*Brassica napus*
Unigene13114_All	87.87	31.7	-1.47	V-H^+ ATPase g1	*Brassica napus*
CL4850.Contig2_All	13.77	0.89	-3.96	V-H^+ ATPase h	*Camelina sativa*
CL1819.Contig2_All	132.35	33.53	-1.98	P-H^+ ATPase	*Arabidopsis thaliana*
Unigene3819_All	12.66	4.73	-1.42	P-Ca^{2+} ATPase 8	*Brassica napus*
Unigene8285_All	13.91	3.35	-2.05	P-Ca^{2+} ATPase 13	*Arabidopsis thaliana*
CL5374.Contig2_All	4.3	0.55	-2.97	P-Ca^{2+} ATPase 11	*Camelina sativa*

Table S5. Differentially expressed genes (DEGs) related to ion transport in shoots of *P. cornutum* after 50 mM NaCl treatment for 6 h. C6S RPKM and S6S RPKM indicates the RPKM value of a gene in shoots under control condition for 6 h and salt treatment for 6 h respectively. Fold change equals to $\log_2$ (S6S RPKM / C6S RPKM) and indicates the transcript abundance change of each DEGs. Protein is the protein encoded by each DEGs. The Gene ID of each upregulated DEGs with no expression under control condition is in bold.

Gene ID	C6S RPKM	S6S RPKM	Log_2 Ratio	Protein	Most homologous species
CL4002.Contig2_All	1.6	4.04	1.34	CCC1	*Camelina sativa*
**CL2577.Contig2_All**	0.01	2.52	7.98	CLCf	*Camelina sativa*
**CL4502.Contig3_All**	0.01	2.0	7.69	CLCb	*Camelina sativa*
CL2477.Contig4_All	2.98	15.98	2.42	CLCg	*Camelina sativa*
CL4502.Contig2_All	5.7	0.01	-9.15	CLCa	*Arabidopsis thaliana*
**Unigene7084_All**	0.01	1.44	7.17	NPF5.2	*Eutrema salsugineum*
**CL3282.Contig2_All**	0.01	1.35	7.08	NPF6.2b	*Eutrema salsugineum*
**CL1188.Contig1_All**	0.01	0.13	3.70	NPF8.4	*Eutrema salsugineum*
CL8124.Contig3_All	2.41	15.89	2.72	NPF6.3b	*Eutrema salsugineum*
**CL1892.Contig6_All**	0.01	0.05	2.32	NPF2.11	*Arabidopsis thaliana*
CL3282.Contig1_All	6.9	33.19	2.27	NPF6.2a	*Eutrema salsugineum*
CL2487.Contig3_All	0.38	1.57	2.05	NPF5.9b	*Eutrema salsugineum*
CL1209.Contig3_All	4.02	10.77	1.42	NPF2.13	*Brassica napus*
CL6278.Contig1_All	2.29	5.41	1.24	NPF8.1a	*Eutrema salsugineum*
CL2487.Contig1_All	1.65	3.71	1.17	NPF5.9a	*Eutrema salsugineum*
Unigene30347_All	1.04	2.12	1.03	NPF4.7	*Eutrema salsugineum*
Unigene13454_All	4.08	1.57	-1.38	NPF8.2	*Eutrema salsugineum*
CL5549.Contig1_All	1.2	0.37	-1.70	NPF2.7	*Arabidopsis thaliana*
CL10017.Contig3_All	0.52	0.01	-5.70	NPF5.15	*Eutrema salsugineum*
CL2487.Contig2_All	1.05	0.01	-6.71	NPF5.9	*Eutrema salsugineum*
Unigene15665_All	2.25	0.01	-7.81	NPF7.3	*Brassica napus*
CL10017.Contig1_All	2.89	0.01	-8.17	NPF5.15	*Eutrema salsugineum*
**CL141.Contig8_All**	0.01	4.35	8.76	SOS1	*Thellungiella halophila*

**CL1096.Contig4_All**	0.01	2.53	7.98	NHX5	*Arabidopsis thaliana*
CL1096.Contig10_All	1.11	2.72	1.29	NHX6	*Arabidopsis thaliana*
CL4173.Contig1_All	7.26	14.76	1.02	NHX2	*Arabidopsis thaliana*
Unigene20453_All	0.22	0.01	-4.46	NHX4	*Eutrema salsugineum*
CL6534.Contig4_All	17.64	6.58	-1.42	NCX	*Arabidopsis thaliana*
CL3604.Contig8_All	0.55	1.61	1.55	GLR3.3a	*Brassica napus*
CL3834.Contig2_All	0.06	0.35	2.54	HKT1	*Brassica napus*
CL1280.Contig5_All	3.98	15.47	1.96	KUP2	*Arabidopsis thaliana*
CL4846.Contig1_All	0.64	1.86	1.54	KT2b	*Brassica napus*
CL2337.Contig1_All	0.96	0.01	-6.58	KUP9	*Camelina sativa*
CL166.Contig1_All	24.6	8.23	-1.58	SKOR	*Arabidopsis thaliana*
CL1585.Contig1_All	2.07	5.44	1.39	KEA6a	*Brassica rapa*
CL1585.Contig4_All	0.72	0.01	-6.17	KEA6b	*Brassica napus*
**CL3663.Contig7_All**	0.01	1.81	7.50	CNGC6a	*Camelina sativa*
Unigene6900_All	0.4	1.79	2.16	CNGC1b	*Arabidopsis thaliana*
Unigene5024_All	0.75	1.86	1.31	CNGC1a	*Arabidopsis thaliana*
CL1912.Contig1_All	0.59	0.01	-5.88	V-CHX18	*Arabidopsis thaliana*
Unigene16699_All	0.61	3.02	2.31	Sultr1.2b	*Brassica rapa*
CL7451.Contig1_All	3.57	7.36	1.04	Sultr2.2	*Brassica rapa*
CL7423.Contig3_All	0.91	4.04	2.15	ZnT	*Camelina sativa*
CL9830.Contig1_All	2.49	5.84	1.23	ZnT	*Arabidopsis thaliana*
**Unigene4968_All**	0.01	18.61	10.86	CTR5a	*Brassica napus*
Unigene13264_All	1.81	6.66	1.88	CTR5b	*Brassica napus*
CL8249.Contig2_All	1.31	3.79	1.53	CTR4	*Brassica napus*
Unigene6326_All	29.25	69.68	1.25	CTR1c	*Brassica rapa*
Unigene3070_All	7.24	14.5	1.00	CTR1b	*Brassica rapa*
Unigene29796_All	21.99	6.49	-1.76	CTR5c	*Brassica napus*
CL2740.Contig2_All	7.97	3.06	-1.38	MnT PDR2	*Camelina sativa*
**CL7845.Contig1_All**	0.01	1.13	6.82	MGT	*Arabidopsis thaliana*
Unigene1617_All	0.63	3.27	2.38	MGT	*Arabidopsis thaliana*
CL2740.Contig1_All	4.97	10.65	1.10	MGT	*Camelina sativa*
CL6691.Contig2_All	81.69	23.51	-1.80	AMT1.1	*Camelina sativa*
CL6691.Contig1_All	2.88	0.76	-1.92	AMT1.2	*Arabidopsis thaliana*
CL1590.Contig1_All	20.13	4.91	-2.04	AMT2	*Brassica rapa*
**CL6281.Contig2_All**	0.01	6.75	9.40	V-H^+ ATPase e1	*Arabidopsis thaliana*
CL2339.Contig1_All	0.24	3.49	3.86	V-H^+ ATPase c1	*Camelina sativa*
Unigene3164_All	69.02	32.95	-1.07	V-H^+ ATPase f	*Camelina sativa*
CL9511.Contig2_All	10.58	4.56	-1.21	V-H^+ ATPase c2	*Arabidopsis thaliana*
CL4850.Contig2_All	0.82	0.01	-6.36	V-H^+ ATPase h	*Brassica napus*
CL1819.Contig2_All	0.27	10.36	5.26	P-H^+ ATPase	*Arabidopsis thaliana*
CL1141.Contig1_All	11.3	24.38	1.11	P-H^+ ATPase 2	*Camelina sativa*
Unigene15025_All	22.37	47.16	1.08	P-H^+ ATPase 3	*Brassica napus*
CL3452.Contig2_All	0.06	3.78	5.98	P-Ca^{2+} ATPase 1	*Arabidopsis thaliana*
CL972.Contig1_All	1.98	0.3	-2.72	P-Ca^{2+} ATPase 10	*Brassica rapa*
Unigene8285_All	2.35	0.09	-4.71	P-Ca^{2+} ATPase 13	*Arabidopsis thaliana*

Table S6. Differentially expressed genes (DEGs) related to ion transport in shoots of *P. cornutum* after 50 mM NaCl treatment for 24 h. C24S RPKM and S24S RPKM indicates the RPKM value of a gene in shoots under control condition for 24 h and salt treatment for 24 h respectively. Fold change equals to $\log_2$ (S24S RPKM / C24S RPKM) and indicates the transcript abundance change of each DEGs. Protein is the protein encoded by each DEGs. The Gene ID of each upregulated DEGs with no expression under control condition is in bold.

Gene ID	C24S RPKM	S24S RPKM	Log_2 Ratio	Protein	Most homologous species
**CL4002.Contig2_All**	0.01	1.5	7.23	CCC1	*Camelina sativa*
CL2577.Contig3_All	4.83	16.23	1.75	CLCf	*Camelina sativa*

CL2477.Contig4_All	9.68	21.99	1.18	CLCg	*Camelina sativa*
**Unigene7084_All**	0.01	1.95	7.61	NPF5.2	*Eutrema salsugineum*
**CL3282.Contig2_All**	0.01	1.31	7.03	NPF6.2b	*Eutrema salsugineum*
**CL6278.Contig1_All**	0.01	0.49	5.61	NPF8.1a	*Eutrema salsugineum*
**CL568.Contig1_All**	0.01	0.05	2.32	NPF2.1	*Brassica napus*
CL2487.Contig3_All	0.98	3.07	1.65	NPF5.9b	*Eutrema salsugineum*
CL4142.Contig1_All	3.37	6.87	1.03	NPF7.3	*Brassica napus*
CL1188.Contig4_All	1.91	0.9	-1.09	NPF8.4	*Eutrema salsugineum*
CL10017.Contig1_All	2.08	0.89	-1.22	NPF5.15	*Eutrema salsugineum*
CL5549.Contig1_All	0.09	0.01	-3.17	NPF2.7a	*Arabidopsis thaliana*
Unigene12441_All	0.27	0.01	-4.75	NPF2.12	*Raphanus sativus*
CL3477.Contig1_All	0.28	0.01	-4.81	NPF4.4	*Eutrema salsugineum*
CL5549.Contig2_All	0.44	0.01	-5.46	NPF2.7b	*Arabidopsis thaliana*
**CL1096.Contig10_All**	0.01	1.08	6.75	NHX6	*Eutrema salsugineum*
Unigene7147_All	0.43	1.25	1.54	NHX1	*Capsella rubella*
CL1096.Contig4_All	2.18	4.57	1.07	NHX5	*Arabidopsis thaliana*
CL141.Contig1_All	0.23	0.01	-4.52	NHX8	*Eutrema salsugineum*
CL2417.Contig1_All	3.05	13.97	2.20	NCX3	*Arabidopsis thaliana*
CL6534.Contig4_All	7.68	2.62	-1.55	NCX1	*Arabidopsis thaliana*
CL7855.Contig1_All	3.51	1.52	-1.21	GLR1.2	*Camelina sativa*
CL66.Contig2_All	2.47	0.87	-1.51	GLR3.4	*Camelina sativa*
CL415.Contig2_All	1.43	0.39	-1.87	GLR3.2	*Arabidopsis thaliana*
CL3604.Contig6_All	1.1	0.01	-6.78	GLR3.3a	*Brassica napus*
**CL3834.Contig2_All**	0.01	1.51	7.24	HKT1	*Brassica napus*
CL1280.Contig6_All	0.51	1.54	1.59	KUP2	*Arabidopsis thaliana*
CL5926.Contig1_All	1.29	2.62	1.02	KT2a	*Brassica napus*
Unigene5974_All	0.59	0.19	-1.63	KT3	*Eutrema salsugineum*
CL5926.Contig2_All	2.39	0.01	-7.90	KT2b	*Brassica napus*
CL2337.Contig9_All	5.76	0.01	-9.17	KUP9	*Camelina sativa*
CL1599.Contig2_All	1.76	5.56	1.66	KEA4	*Arabidopsis thaliana*
CL1585.Contig6_All	0.74	0.01	-6.21	KEA6	*Brassica napus*
CL166.Contig1_All	13.17	3.14	-2.07	SKOR	*Arabidopsis thaliana*
**CL9004.Contig2_All**	0.01	3.37	8.40	GORK	*Arabidopsis thaliana*
**Unigene12647_All**	0.01	0.43	5.43	CNGC8b	*Eutrema salsugineum*
Unigene6900_All	0.25	0.82	1.71	CNGC1b	*Arabidopsis thaliana*
Unigene5024_All	1.77	0.78	-1.18	CNGC1a	*Arabidopsis thaliana*
Unigene6260_All	0.83	0.31	-1.42	CNGC14	*Eutrema salsugineum*
Unigene1860_All	0.18	0.01	-4.17	CNGC8a	*Eutrema salsugineum*
CL3663.Contig7_All	1.36	0.01	-7.09	CNGC6a	*Camelina sativa*
CL1894.Contig2_All	1.49	0.4	-1.90	Sultr3.4	*Camelina sativa*
CL7314.Contig1_All	3.03	8.07	1.41	ZnT	*Arabidopsis thaliana*
CL7423.Contig3_All	0.91	0.01	-6.51	ZnT	*Camelina sativa*
**Unigene13264_All**	0.01	4.84	8.92	CTR5b	*Brassica napus*
Unigene29796_All	1.69	7.64	2.18	CTR5c	*Brassica napus*
CL8249.Contig1_All	4.25	9.58	1.17	CTR1a	*Brassica napus*
Unigene4968_All	9.06	0.01	-9.82	CTR5a	*Brassica napus*
**CL2740.Contig2_All**	0.01	5.34	9.06	MnT PDR2	*Camelina sativa*
**Unigene1619_All**	0.01	1.67	7.38	MGT10b	*Arabidopsis thaliana*
Unigene1617_All	0.66	3.22	2.29	MGT10a	*Arabidopsis thaliana*
CL1590.Contig1_All	2.43	0.15	-4.02	AMT2	*Brassica rapa*
**Unigene17151_All**	0.01	2.34	7.87	V-H^+ ATPase c2	*Camelina sativa*
**CL4850.Contig2_All**	0.01	1.95	7.61	V-H^+ ATPase h	*Brassica napus*
CL972.Contig1_All	0.3	1.37	2.19	P-Ca^{2+} ATPase 10	*Brassica rapa*
CL3452.Contig4_All	15.26	3.03	-2.33	P-Ca^{2+} ATPase 1	*Arabidopsis thaliana*

Table S7. Differentially expressed genes (DEGs) related to ROS-scavenging system in roots of *P. cornutum* after 50 mM NaCl treatment for 6 h. C6R RPKM and S6R RPKM indicates the

RPKM value of a gene in roots under control condition for 6 h and salt treatment for 6 h respectively. Fold change equals to log_2 (S6R RPKM / C6R RPKM) and indicates the transcript abundance change of each DEGs. Protein is the protein encoded by each DEGs. The Gene ID of each upregulated DEGs with no expression under control condition is in bold.

Gene ID	C6R RPKM	S6R RPKM	Log_2 Ratio	Protein	Most homologous species
Unigene1431_All	33.27	69.27	1.06	GLR-C1	*Eutrema salsugineum*
Unigene5963_All	191.25	68.67	-1.48	GLR-S13	*Arabidopsis thaliana*
**CL887.Contig5_All**	0.01	1.23	6.94	APX5	*Arabidopsis thaliana*
Unigene8734_All	24.62	55.94	1.18	APX1	*Arabidopsis thaliana*
**CL110.Contig1_All**	0.01	11.77	10.20	GST-U10	*Arabidopsis lyrata*
CL2973.Contig2_All	1.44	58.87	5.35	GST-F3	*Arabidopsis thaliana*
CL5907.Contig2_All	0.76	4.52	2.57	GST-U5	*Arabidopsis thaliana*
CL2973.Contig4_All	39.43	98.6	1.32	GST-F6a	*Brassica oleracea*
CL5128.Contig1_All	1.82	4.36	1.26	GST-F2	*Brassica napus*
Unigene11304_All	7.73	18.03	1.22	GST-U28	*Arabidopsis lyrata*
CL9702.Contig2_All	36.46	78.01	1.10	GST-L1	*Arabidopsis thaliana*
Unigene10083_All	76.97	164.29	1.09	GST-U12	*Brassica oleracea*
Unigene29119_All	163.4	344.2	1.07	GST-F6b	*Brassica oleracea*
Unigene9630_All	23.9	9.94	-1.27	GST-F11	*Arabidopsis thaliana*
Unigene11523_All	159.8	60.18	-1.41	GST-U19	*Arabidopsis thaliana*
CL4061.Contig2_All	1.59	4.13	1.38	GPX1b	*Brassica napus*
CL4061.Contig1_All	7.03	3.33	-1.08	GPX1a	*Brassica napus*
**CL2989.Contig3_All**	0.01	1.43	7.16	PEX3-1a	*Arabidopsis thaliana*
CL9369.Contig2_All	6.84	18.4	1.43	PEX11e	*Arabidopsis thaliana*
CL9371.Contig1_All	0.72	3.72	2.37	Trxh10	*Eutrema salsugineum*
Unigene16702_All	77.29	248.91	1.69	Trxh8	*Eutrema salsugineum*
CL1304.Contig3_All	3.68	11.76	1.68	NTrx3	*Eutrema salsugineum*
CL8308.Contig3_All	5.46	15.37	1.49	Trxm2	*Eutrema salsugineum*
CL5332.Contig5_All	0.63	0.01	-5.98	Trxh9	*Eutrema salsugineum*

Table S8. Differentially expressed genes (DEGs) related to ROS-scavenging system in roots of *P. cornutum* after 50 mM NaCl treatment for 24 h. C24R RPKM and S24R RPKM indicates the RPKM value of a gene in roots under control condition for 24 h and salt treatment for 24 h respectively. Fold change equals to log_2 (S24R RPKM / C24R RPKM) and indicates the transcript abundance change of each DEGs. Protein is the protein encoded by each DEGs. The Gene ID of each upregulated DEGs with no expression under control condition is in bold.

Gene ID	C24R RPKM	S24R RPKM	Log_2 Ratio	Protein	Most homologous species
Unigene18864_All	1.79	6.82	1.93	GLR-C8	*Camelina sativa*
CL887.Contig5_All	1.23	0.01	-6.94	APX5	*Arabidopsis thaliana*
CL4446.Contig2_All	2.78	0.01	-8.12	APX6	*Arabidopsis thaliana*
**CL110.Contig1_All**	0.01	13.2	10.37	GST-U10	*Arabidopsis thaliana*
Unigene18400_All	7.11	84.14	3.56	GST-F5	*Arabidopsis thaliana*
CL6349.Contig3_All	65.73	245.7	1.90	GST-R1	*Arabidopsis thaliana*
Unigene9630_All	5.87	15.79	1.43	GST-F11	*Arabidopsis thaliana*
CL7899.Contig2_All	14.32	31.19	1.12	GST-U22	*Arabidopsis thaliana*
Unigene19878_All	20.9	10.08	-1.05	GST-U21	*Arabidopsis lyrata*
Unigene5105_All	100.27	47.54	-1.08	GST-U7	*Arabidopsis thaliana*
Unigene19876_All	64.42	8.48	-2.93	GST-U11b	*Arabidopsis lyrata*
CL7537.Contig1_All	179.44	23.06	-2.96	GST-U11a	*Brassica napus*
Unigene5116_All	12.11	0.62	-4.29	GST-U4	*Arabidopsis thaliana*
Unigene5565_All	3.49	0.01	-8.45	GST-U18	*Arabidopsis thaliana*
CL9702.Contig3_All	83.27	0.01	-13.02	GST-L1	*Arabidopsis thaliana*
CL3965.Contig2_All	1.86	0.13	-3.84	GPX2	*Arabidopsis thaliana*

CL2179.Contig1_All	105.8	35.25	-1.59	GPX6	*Arabidopsis thaliana*
Unigene8465_All	5.69	1.92	-1.57	GPX3	*Camelina sativa*
CL3935.Contig2_All	3.9	13.51	1.79	POD7	*Brassica rapa*
CL1020.Contig1_All	2.39	7.11	1.57	POD34	*Raphanus sativus*
CL1440.Contig4_All	1.53	0.01	-7.26	POD53	*Arabidopsis lyrata*
Unigene19077_All	24.72	3.78	-2.71	POD22	*Raphanus sativus*
CL6178.Contig2_All	6.4	1.48	-2.11	POD11	*Arabidopsis lyrata*
Unigene20272_All	9.27	2.56	-1.86	POD44	*Camelina sativa*
Unigene372_All	13.71	4.27	-1.68	POD17	*Brassica rapa*
CL1299.Contig3_All	5.2	2.04	-1.35	POD2a	*Arabidopsis thaliana*
CL9448.Contig3_All	123.09	54.44	-1.18	POD37	*Arabidopsis thaliana*
Unigene21509_All	118.13	57.05	-1.05	POD56	*Arabidopsis thaliana*
CL9260.Contig1_All	155.12	75.65	-1.04	POD52	*Arabidopsis thaliana*
Unigene724_All	34.57	17.04	-1.02	POD2b	*Arabidopsis thaliana*
CL1859.Contig3_All	336.7	787.37	1.23	CAT3	*Arabidopsis thaliana*
Unigene2251_All	66.16	12.88	-2.36	CAT2	*Raphanus sativus*
**CL2989.Contig6_All**	0.01	2.22	7.79	PEX3-1a	*Arabidopsis thaliana*
CL2989.Contig4_All	2.27	0.01	-7.83	PEX3-1b	*Arabidopsis thaliana*
CL511.Contig8_All	0.8	0.01	-6.32	PEX2	*Arabidopsis lyrata*
**CL3847.Contig3_All**	0.01	5.54	9.11	Trxf1	*Arabidopsis thaliana*
**CL5332.Contig7_All**	0.01	2.39	7.90	Trxh9	*Arabidopsis thaliana*
**CL7421.Contig1_All**	0.01	1.43	7.16	Trxm4	*Arabidopsis lyrata*
Unigene16702_All	47.13	112.49	1.26	Trxh8	*Raphanus sativus*
CL1304.Contig4_All	12.68	4.74	-1.42	NTrx3	*Arabidopsis thaliana*
Unigene14588_All	7.71	2.23	-1.79	Trxm3	*Arabidopsis thaliana*
Unigene8256_All	5.41	1.51	-1.84	Trxh9	*Arabidopsis thaliana*
CL4087.Contig2_All	3.76	13.58	1.85	SOD Cu-Zn	*Arabidopsis thaliana*
Unigene9945_All	11.59	3.25	-1.83	SOD Mn	*Arabidopsis thaliana*

Table S9. Differentially expressed genes (DEGs) related to ROS-scavenging system in shoots of *P. cornutum* after 50 mM NaCl treatment for 6 h. C6S RPKM and S6S RPKM indicates the RPKM value of a gene in shoots under control condition for 6 h and salt treatment for 6 h respectively. Fold change equals to $\log_2$ (S6S RPKM / C6S RPKM) and indicates the transcript abundance change of each DEGs. Protein is the protein encoded by each DEGs. The Gene ID of each upregulated DEGs with no expression under control condition is in bold.

Gene ID	C6S RPKM	S6S RPKM	Log_2 Ratio	Protein	Most homologous species
Unigene3075_All	57.12	5.9	-3.28	GLR-C9	*Arabidopsis lyrata*
CL7050.Contig3_All	5.47	2.03	-1.43	GLR-C5	*Arabidopsis thaliana*
Unigene8088_All	7.62	3.17	-1.27	GLR-S11	*Brassica napus*
CL4446.Contig2_All	26.03	5.12	-2.35	APX6	*Arabidopsis thaliana*
**CL767.Contig4_All**	0.01	6.41	9.32	GST-F9	*Arabidopsis thaliana*
L2973.Contig3_All	0.61	2.86	2.23	GST-F3	*Arabidopsis thaliana*
Unigene8549_All	8.77	20.86	1.25	GST-U24	*Arabidopsis thaliana*
CL8158.Contig2_All	106.21	31.75	-1.74	GST-U12	*Arabidopsis thaliana*
Unigene16310_All	4.31	0.12	-5.17	GST-F12	*Arabidopsis thaliana*
Unigene14577_All	1.48	0.01	-7.21	GST-F7	*Arabidopsis thaliana*
CL4061.Contig2_All	2.01	4.73	1.23	GPX1	*Arabidopsis thaliana*
CL3965.Contig1_All	6.29	2.23	-1.50	GPX2	*Arabidopsis thaliana*
**CL3093.Contig7_All**	0.01	1.73	7.43	POD43	*Arabidopsis lyrata*
CL2485.Contig1_All	2.48	7.48	1.59	POD44	*Arabidopsis lyrata*
Unigene6483_All	12.5	3.87	-1.69	POD12	*Arabidopsis lyrata*
CL7594.Contig2_All	4.3	0.23	-4.22	POD71	*Arabidopsis lyrata*
CL1440.Contig1_All	2.27	0.01	-7.83	POD2	*Arabidopsis thaliana*
**CL2989.Contig1_All**	0.01	1.48	7.21	PEX3-1a	*Arabidopsis thaliana*
CL9369.Contig2_All	5.07	10.39	1.04	PEX11E	*Arabidopsis thaliana*

**CL9371.Contig2_All**	0.01	2.02	7.66	Trx4	*Arabidopsis lyrata*
**CL270.Contig3_All**	0.01	1.79	7.48	Trx-O2	*Arabidopsis thaliana*
CL3202.Contig3_All	0.56	2.7	2.27	Trx1	*Arabidopsis lyrata*
Unigene9687_All	2.42	7.56	1.64	Trx2b	*Capsella rubella*
CL1304.Contig4_All	0.68	1.67	1.30	NTrx3	*Eutrema salsugineum*
CL8308.Contig3_All	126.58	269.65	1.09	Trx-M2	*Eutrema salsugineum*
CL9371.Contig1_All	3.9	0.01	-8.61	Trx6	*Eutrema salsugineum*
CL6394.Contig6_All	9.03	3.26	-1.47	Trx2a	*Eutrema salsugineum*
Unigene12562_All	9.52	66.03	2.80	2-Cys PrxR B	*Eutrema salsugineum*

Table S10. Differentially expressed genes (DEGs) related to ROS-scavenging system in shoots of *P. cornutum* after 50 mM NaCl treatment for 24 h. C24S RPKM and S24S RPKM indicates the RPKM value of a gene in shoots under control condition for 24 h and salt treatment for 24 h respectively. Fold change equals to $\log_2$(S24S RPKM / C24S RPKM) and indicates the transcript abundance change of each DEGs. Protein is the protein encoded by each DEGs. The Gene ID of each upregulated DEGs with no expression under control condition is in bold.

**Gene ID**	**C24S RPKM**	**S24S RPKM**	**$\mathbf{Log_2}$ Ratio**	**Protein**	**Most homologous species**
Unigene3075_All	34.1	4.18	3	GLR-C9	*Arabidopsis lyrata*
CL2770.Contig1_All	5.84	13.96	1.26	APX1	*Arabidopsis thaliana*
CL4446.Contig2_All	13.02	4.99	-1.38	APX6	*Arabidopsis thaliana*
CL2261.Contig1_All	0.97	3.33	1.78	GST-L3	*Arabidopsis thaliana*
Unigene9682_All	6.16	2.34	-1.40	GST-U13	*Arabidopsis thaliana*
Unigene20207_All	14.06	5.18	-1.44	GST-U22	*Arabidopsis thaliana*
Unigene8549_All	15.75	4.71	-1.74	GST-U24	*Arabidopsis thaliana*
CL3965.Contig1_All	2.17	0.01	-7.76	GPX2	*Arabidopsis thaliana*
CL7594.Contig2_All	2.16	0.16	-3.75	POD71	*Eutrema salsugineum*
CL1859.Contig3_All	927.78	2016.81	1.12	CAT3	*Eutrema salsugineum*
**CL2989.Contig1_All**	0.01	2.31	7.85	PEX3-1a	*Arabidopsis thaliana*
CL2989.Contig3_All	4.08	1.75	-1.22	PEX3-1b	*Eutrema salsugineum*
CL7826.Contig1_All	1.51	5.86	1.96	Trx-S8	*Eutrema salsugineum*
CL8397.Contig2_All	4.55	9.84	1.11	Trx3-3	*Eutrema salsugineum*
CL9646.Contig1_All	2.39	7.32	1.61	Trx3-2	*Eutrema salsugineum*
**CL4050.Contig1_All**	0.01	3.31	8.37	SOD Cu-Zn	*Eutrema salsugineum*
Unigene9945_All	20.71	0.73	-4.83	SOD Mn	*Eutrema salsugineum*

Table S11. Differentially expressed genes (DEGs) related to photosynthesis in shoots of *P. cornutum* after 50 mM NaCl treatment for 6 h. C6S RPKM and S6S RPKM indicates the RPKM value of a gene in shoots under control condition for 6 h and salt treatment for 6 h respectively. Fold change equals to log_2 (S6S RPKM / C6S RPKM) and indicates the transcript abundance change of each DEGs. Protein is the protein encoded by each DEGs. The Gene ID of each upregulated DEGs with no expression under control condition is in bold.

	Gene ID	C6S RPKM	S6S RPKM	Log_2 Ratio	Protein	Most homologous species
Chlorophyll biosynthesis	**CL5192.Contig2_All**	0.01	1.33	7.06	protoporphyrinogen oxidase 2	*Capsella rubella*
	**CL6305.Contig1_All**	0.01	0.96	6.58	uroporphyrinogen-III C-methyltransferase	*Brassica rapa*
	CL6357.Contig2_All	0.3	2.32	2.95	uroporphyrinogen-III C-methyltransferase	*Arabidopsis lyrata*
	CL1.Contig6_All	0.5	2.73	2.45	uroporphyrinogen-III synthase	*Eutrema salsugineum*
	CL1607.Contig4_All	17.49	47.94	1.45	uroporphyrinogen decarboxylase 1	*Eutrema salsugineum*
	Unigene18440_All	0.48	0.01	-5.58	protoporphyrinogen oxidase 1	*Eutrema salsugineum*
	CL3982.Contig3_All	0.85	0.01	-6.41	protoporphyrinogen oxidase 2	*Eutrema salsugineum*
Chlorophyll catabolism	**CL6260.Contig2_All**	0.01	0.27	4.75	chlorophyll catabolite reductase	*Eutrema salsugineum*
Photosystem II	**Unigene29512_All**	0.01	5.08	8.99	oxygen-evolving enhancer protein 3-1	*Raphanus sativus*
	Unigene31068_All	1.08	10.82	3.32	CP43 chlorophyll apoprotein	*Eutrema salsugineum*
	Unigene5160_All	9.83	74.71	2.93	CP47 chlorophyll apoprotein	*Eutrema salsugineum*
	CL3345.Contig2_All	2.17	6.38	1.56	reaction center PsbP family protein	*Capsella rubella*
	CL5255.Contig3_All	12.15	26.58	1.13	LOW PSII ACCUMULATION 1	*Camelina sativa*
	Unigene29750_All	201.87	84.27	-1.26	oxygen-evolving enhancer protein 2-1	*Eutrema salsugineum*
	Unigene15722_All	328.23	134.3	-1.29	chlorophyll a-b binding protein 1	*Capsella rubella*
	CL877.Contig2_All	7.5	0.01	-9.55	chlorophyll a-b binding protein 5	*Capsella rubella*
Photosystem I	CL874.Contig1_All	0.27	3.55	3.72	P700 chlorophyll a apoprotein A1	*Pugionium dolabratum*
	Unigene30660_All	1.74	17.8	3.35	P700 chlorophyll a apoprotein A2	*Pugionium dolabratum*
	Unigene17981_All	2.02	9.29	2.20	P700 chlorophyll a apoprotein A1	*Pugionium dolabratum*
Cytochrome b6/f	CL2412.Contig4_All	0.02	0.4	4.32	cytochrome b6	*Pugionium dolabratum*
	Unigene30216_All	0.11	0.45	2.03	cytochrome f	*Pugionium dolabratum*
Ferredoxin	CL1825.Contig7_All	1.44	5.45	1.92	ferredoxin-dependent glutamate synthase 2	*Raphanus sativus*
	CL6172.Contig3_All	2.74	0.86	-1.67	ferredoxin-$NADP^+$ reductase	*Arabidopsis lyrata*
Carbon fixation	**Unigene9951_All**	0.01	14.02	10.45	ribulose-bisphosphate carboxylase	*Pugionium dolabratum*
	**CL4472.Contig3_All**	0.01	2.34	7.87	2-phosphoglycerate kinase	*Arabidopsis lyrata*
	**Unigene30776_All**	0.01	0.94	6.55	2-phosphoglycerate kinase	*Arabidopsis lyrata*
	**Unigene25114_All**	0.01	0.64	6	NADP-dependent malic enzyme 1	*Eutrema salsugineum*
	CL4773.Contig6_All	0.46	4.46	3.28	NADP-dependent malic enzyme 3	*Eutrema salsugineum*
	Unigene265_All	0.12	0.5	2.06	NADP-dependent malic enzyme 1	*Camelina sativa*
	CL3053.Contig5_All	4.08	15.87	1.96	malate dehydrogenase	*Eutrema salsugineum*
	CL4623.Contig2_All	1.01	3.71	1.88	NADP-dependent malic enzyme 2	*Arabidopsis Iyrata*
	CL7513.Contig4_All	27.1	90.55	1.74	triosephosphate isomerase	*Eutrema salsugineum*
	Unigene10475_All	41.2	135.81	1.72	NADP-dependent malic enzyme 4	*Capsella rubella*
	CL3695.Contig2_All	2.19	5.91	1.43	alanine-glyoxylate transaminase 2	*Eutrema salsugineum*
	CL3851.Contig1_All	10.06	24.25	1.27	phosphoglycerate kinase 1	*Brassica napus*
	CL670.Contig5_All	3.49	7.94	1.19	alanine aminotransferase 1	*Eutrema salsugineum*
	CL10208.Contig2_All	2.42	5.18	1.10	phosphoenolpyruvate carboxylase 1	*Eutrema salsugineum*
	Unigene5231_All	0.39	0.83	1.09	NADP-dependent malic enzyme 1	*Arabidopsis lyrata*
	Unigene10001_All	2.33	4.76	1.03	phosphoglycerate kinase	*Eutrema salsugineum*
	CL429.Contig4_All	0.71	1.44	1.02	glyceraldehyde-3-phosphate dehydrogenase	*Eutrema salsugineum*
	Unigene6383_All	376	3	1	glyceraldehyde-3-phosphate dehydrogenase 2	*Eutrema salsugineum*
	CL8434.Contig1_All	5.73	2.78	-1.04	glyceraldehyde-3-phosphate dehydrogenase 2	*Arabidopsis lyrata*
	CL7406.Contig1_All	59.46	25.33	-1.23	phosphoenolpyruvate carboxykinase 1	*Arabidopsis thaliana*
	CL6324.Contig2_All	19.03	7.58	-1.33	ribulose-1,5 bisphosphate carboxylase/oxygenase large subunit	*Arabidopsis lyrata*
	CL3106.Contig2_All	317.69	100.34	-1.66	fructose-bisphosphate aldolase 2	*Eutrema salsugineum*
	Unigene753_All	0.65	0.01	-6.02	2-phosphoglycerate kinase	*Camelina sativa*

	CL7058.Contig1_All	4.23	0.01	-8.72	ribulose-1,5 bisphosphate carboxylase/oxygenase large subunit	*Eutrema salsugineum*
ATP synthease	CL9868.Contig1_All	0.04	0.42	3.39	H^+-transporting ATP synthase subunit a	*Arabidopsis thaliana*
	**Unigene21905_All**	0.01	0.06	2.58	H^+-transporting ATP synthase subunit alpha	*Arabidopsis thaliana*
	Unigene10005_All	0.12	0.62	2.37	H^+-transporting ATP synthase subunit b	*Arabidopsis thaliana*
	Unigene30032_All	1.34	3.83	1.52	H^+-transporting ATP synthase subunit beta	*Arabidopsis thaliana*
	CL1597.Contig1_All	7.76	2.34	1.05	H^+-transporting ATP synthase subunit e	*Arabidopsis thaliana*

Table S12. Differentially expressed genes (DEGs) related to photosynthesis in shoots of *P. cornutum* after 50 mM NaCl treatment for 24 h. C24S RPKM and S24S RPKM indicates the RPKM value of a gene in shoots under control condition for 24 h and salt treatment for 24 h respectively. Fold change equals to $\log_2$ (S24S RPKM / C24S RPKM) and indicates the transcript abundance change of each DEGs. Protein is the protein encoded by each DEGs. The Gene ID of each upregulated DEGs with no expression under control condition is in bold.

	Gene ID	C24S RPKM	S24S RPKM	Log_2 Ratio	Protein	Most homologous species
Chlorophyll biosynthesis	**CL4285.Contig3_All**	0.01	1.09	6.77	coproporphyrinogen-III oxidase 1	*Arabidopsis lyrata*
	**CL3982.Contig4_All**	0.01	0.67	6.07	protoporphyrinogen oxidase 2	*Eutrema salsugineum*
	CL1607.Contig3_All	2.09	18.65	3.16	uroporphyrinogen decarboxylase 1	*Eutrema salsugineum*
	CL6357.Contig2_All	1.52	3.44	1.18	uroporphyrinogen III methyltransferase	*Arabidopsis thaliana*
	CL1.Contig9_All	0.86	0.09	-3.26	uroporphyrinogen-III synthase	*Eutrema salsugineum*
Chlorophyll catabolism	**CL6260.Contig2_All**	0.01	0.3	4.91	chlorophyll catabolite reductase	*Eutrema salsugineum*
Photosystem II	**CL5140.Contig1_All**	0.01	0.9	6.49	oxygen-evolving enhancer protein 2-1	*Eutrema salsugineum*
	CL877.Contig2_All	2.54	13.19	2.38	chlorophyll a-b binding protein 26	*Eutrema salsugineum*
	Unigene30458_All	0.8	0.39	-1.04	oxygen-evolving enhancer protein 3	*Pugionium dolabratum*
	CL3690.Contig2_All	17.17	5.38	-1.67	reaction center Psb Protein 4	*Eutrema salsugineum*
	Unigene30695_All	13.24	3.34	-1.99	CP47 chlorophyll apoprotein	*Pugionium dolabratum*
	CL9163.Contig1_All	4.35	0.88	-2.31	reaction center Psb Protein	*Pugionium dolabratum*
	Unigene5160_All	61.78	10.87	-2.51	P680 reaction center D1 protein	*Pugionium dolabratum*
	Unigene31068_All	19	1.31	-3.86	CP43 chlorophyll apoprotein	*Pugionium dolabratum*
	Unigene30208_All	1.96	0.09	-4.44	cytochrome b559 subunit alpha	*Pugionium dolabratum*
	CL5255.Contig1_All	4.06	0.01	-8.67	LOW PSII ACCUMULATION 1	*Arabidopsis lyrata*
Photosystem I	CL1039.Contig2_All	2.07	4.47	1.11	photosystem I subunit K	*Arabidopsis thaliana*
	Unigene13985_All	6.21	1.44	-2.11	P700 chlorophyll a apoprotein A1	*Pugionium dolabratum*
	Unigene30660_All	33.67	1.77	-4.25	P700 chlorophyll a apoprotein A2	*Pugionium dolabratum*
	Unigene31256_All	0.22	0.01	-4.46	photosystem I assembly protein Ycf3	*Arabidopsis thaliana*
	CL874.Contig1_All	6.11	0.25	-4.61	P700 chlorophyll a apoprotein A1	*Pugionium dolabratum*
	Unigene17981_All	31.28	0.91	-5.10	P700 chlorophyll a apoprotein A1	*Pugionium dolabratum*
Cytochrome b6/f	Unigene30216_All	4063	26	3	cytochrome f	*Pugionium dolabratum*
	CL2412.Contig4_All	2.9	1.05	-1.47	cytochrome b6	*Pugionium dolabratum*
Ferredoxin	**CL6172.Contig2_All**	0.01	0.94	6.55	ferredoxin-$NADP^+$ reductase	*Arabidopsis lyrata*
	**CL5399.Contig2_All**	0.01	0.31	4.95	ferredoxin oxidoreductase	*Brassica napus*
	CL1825.Contig7_All	1.19	4.01	1.75	ferredoxin-dependent glutamate synthase 2	*Raphanus sativus*
Carbon fixation	**CL429.Contig6_All**	0.01	45.2	12.14	glyceraldehyde-3-phosphate dehydrogenase	*Eutrema salsugineum*
	**CL1969.Contig2_All**	0.01	2.31	7.85	alanine aminotransferase 1	*Eutrema salsugineum*
	**Unigene265_All**	0.01	1.12	6.81	NADP-dependent malic enzyme 1	*Camelina sativa*
	**CL4472.Contig4_All**	0.01	1.07	6.74	2-phosphoglycerate kinase	*Arabidopsis lyrata*
	**Unigene26264_All**	0.01	0.32	5	fructose-bisphosphate aldolase	*Frankliniella occidentalis*
	**CL4271.Contig1_All**	0.01	0.2	4.32	ribulose-bisphosphate carboxylase large chain	*Pugionium dolabratum*
	**Unigene23280_All**	0.01	0.13	3.70	glyceraldehyde-3-phosphate dehydrogenase 2	*Frankliniella occidentalis*
	Unigene25114_All	0.32	3.45	3.43	NADP-dependent malic enzyme 1	*Eutrema salsugineum*
	Unigene5231_All	0.56	3.13	2.48	NADP-dependent malic enzyme 1	*Arabidopsis lyrata*
	CL3695.Contig2_All	2.27	7.04	1.63	alanine-glyoxylate transaminase 2	*Eutrema salsugineum*

	CL8417.Contig3_All	1.23	3.74	1.60	ribulose-1,5 bisphosphate carboxylase/oxygenase large subunit N-methyltransferase	*Eutrema salsugineum*
	CL6045.Contig4_All	0.49	1.44	1.56	phosphoenolpyruvate carboxykinase	*Brassica napus*
	CL8434.Contig3_All	0.41	1.19	1.54	glyceraldehyde-3-phosphate dehydrogenase 2	*Arabidopsis lyrata*
	CL3851.Contig3_All	1.19	3.39	1.51	phosphoglycerate kinase 1	*Brassica napus*
	CL4623.Contig4_All	0.9	2.43	1.43	NADP-dependent malic enzyme 2	*Arabidopsis Iyrata*
	Unigene30785_All	250.72	610.29	1.28	ribulose bisphosphate carboxylase small chain 1B	*Arabidopsis lyrata*
	CL7513.Contig1_All	9.53	21.08	1.15	triosephosphate isomerase	*Eutrema salsugineum*
	CL3053.Contig5_All	8.39	17.65	1.07	malate dehydrogenase	*Eutrema salsugineum*
	CL2188.Contig3_All	4.34	8.88	1.03	fructose-2,6-bisphosphatase	*Camelina sativa*
	CL7406.Contig2_All	1108	2	1	phosphoenolpyruvate carboxykinase 1	*Arabidopsis thaliana*
	Unigene30776_All	0.67	0.33	-1.02	2-phosphoglycerate kinase	*Arabidopsis lyrata*
	Unigene834_All	2.36	0.92	-1.36	phosphoenolpyruvate carboxykinase 2	*Brassica napus*
	CL4773.Contig6_All	2.5	0.97	-1.37	NADP-dependent malic enzyme 3	*Eutrema salsugineum*
	CL861.Contig4_All	9482.71	2219.23	-2.10	ribulose bisphosphate carboxylase small chain 1B	*Eutrema salsugineum*
	Unigene9041_All	0.57	0.01	-5.83	glyceraldehyde-3-phosphate dehydrogenase 1	*Eutrema salsugineum*
	Unigene9951_All	23.69	0.01	-11.21	ribulose-bisphosphate carboxylase	*Pugionium dolabratum*
ATP synthase	**Unigene18322_All**	0.01	0.28	4.81	H^+-transporting ATPase subunit beta	*Eutrema salsugineum*
	**Unigene16853_All**	0.01	0.24	4.58	H^+-transporting ATPase subunit beta	*Eutrema salsugineum*
	**Unigene16854_All**	0.01	0.07	2.81	H^+-transporting ATPase subunit beta	*Eutrema salsugineum*
	CL1597.Contig2_All	6.87	2.24	-1.62	H^+-transporting ATPase subunit e	*Arabidopsis thaliana*
	Unigene10005_All	0.5	0.16	-1.64	H^+-transporting ATPase subunit b	*Arabidopsis thaliana*
	Unigene5734_All	2.03	0.22	-3.21	H^+-transporting ATPase subunit epsilon	*Arabidopsis thaliana*
	CL9868.Contig1_All	0.88	0.07	-3.65	H^+-transporting ATPase subunit a	*Arabidopsis thaliana*
	CL9157.Contig1_All	11.38	0.01	-10.15	H^+-transporting ATPase subunit gamma	*Arabidopsis thaliana*

Table S13. Differentially expressed genes (DEGs) related to transcript factors in roots of *P. cornutum* after 50 mM NaCl treatment for 6 h. C6R RPKM and S6R RPKM indicates the RPKM value of a gene in shoots under control condition for 6 h and salt treatment for 6 h respectively. Fold change equals to log_2(S6R RPKM / C6R RPKM) and indicates the transcript abundance change of each DEGs. Protein is the protein encoded by each DEGs. The Gene ID of each upregulated DEGs with no expression under control condition is in bold.

Gene ID	C6R RPKM	S6R RPKM	Log_2 Ratio	Protein	Most homologous species
**CL2623.Contig9_All**	0.01	0.79	6.30	WRKY4	*Eutrema salsugineum*
Unigene1949_All	1.38	5.76	2.06	WRKY38	*Arabidopsis thaliana*
CL8480.Contig1_All	4.61	15.62	1.76	WRKY51	*Eutrema salsugineum*
CL9644.Contig1_All	5.36	17.95	1.74	WRKY21	*Eutrema salsugineum*
Unigene9981_All	1.29	4.25	1.72	WRKY33b	*Eutrema salsugineum*
CL8143.Contig1_All	1.66	5.25	1.66	WRKY40	*Camelina sativa*
Unigene20839_All	2.77	8.54	1.62	WRKY71	*Eutrema salsugineum*
CL5153.Contig1_All	3.22	8.35	1.37	WRKY33a	*Eutrema salsugineum*
CL2762.Contig1_All	4.33	10.95	1.34	WRKY2	*Eutrema salsugineum*
Unigene14671_All	13.19	32.86	1.32	WRKY54b	*Raphanus sativus*
Unigene11284_All	35.45	81.49	1.20	WRKY54a	*Arabidopsis lyrata*
CL1359.Contig1_All	6.69	15.3	1.19	WRKY36	*Eutrema salsugineum*
CL6227.Contig1_All	4.66	9.92	1.09	WRKY15	*Eutrema salsugineum*
Unigene19359_All	12.66	25.94	1.03	WRKY14	*Raphanus sativus*
CL2682.Contig2_All	95.97	45.12	-1.09	WRKY11	*Eutrema salsugineum*
CL8454.Contig4_All	12.93	3.33	-1.96	WRKY48	*Arabidopsis lyrata*
Unigene3510_All	4.94	1.11	-2.15	WRKY13	*Eutrema salsugineum*
Unigene20840_All	4.35	0.62	-2.81	WRKY28b	*Eutrema salsugineum*
CL3331.Contig1_All	0.71	0.01	-6.15	WRKY19	*Arabidopsis lyrata*
CL2519.Contig2_All	1.24	0.01	-6.95	WRKY20a	*Arabidopsis lyrata*
Unigene20838_All	4.08	0.01	-8.67	WRKY28a	*Brassica napus*
**CL1196.Contig22_All**	0.01	3.01	8.23	MYB58	*Eutrema salsugineum*
**CL6244.Contig1_All**	0.01	2.24	7.81	MYB122b	*Eutrema salsugineum*
Unigene19694_All	1	4.5	2.17	MYB108	*Raphanus sativus*
CL2438.Contig2_All	7.06	29.31	2.05	MYB-PHL7	*Eutrema salsugineum*
CL9050.Contig1_All	12.59	47.13	1.90	MYB-EFMa	*Camelina sativa*
Unigene19391_All	1.53	5.58	1.87	MYB40	*Arabidopsis thaliana*
CL577.Contig2_All	1.83	6.51	1.83	MYB20	*Eutrema salsugineum*
Unigene29272_All	1.33	4.6	1.79	MYB1R1	*Eutrema salsugineum*
CL9449.Contig2_All	2.6	7.63	1.55	MYB96	*Camelina sativa*
CL3530.Contig3_All	1.98	5.44	1.46	MYB122a	*Arabidopsis lyrata*
CL2633.Contig7_All	10.52	28.63	1.44	MYB48	*Arabidopsis thaliana*
Unigene3660_All	7.44	18.21	1.29	MYB88	*Eutrema salsugineum*
Unigene28974_All	5.81	13.46	1.21	MYB122c	*Arabidopsis lyrata*
Unigene17362_All	15.76	36.18	1.20	MYB3	*Eutrema salsugineum*
CL8127.Contig2_All	19.01	41.06	1.11	MYB36	*Brassica napus*
CL2633.Contig8_All	8.36	16.72	1	MYB59	*Arabidopsis lyrata*
CL5738.Contig2_All	128.94	63.08	-1.03	MYB44	*Eutrema salsugineum*
Unigene13074_All	22.87	10.7	-1.10	MYB-EFMb	*Eutrema salsugineum*
Unigene20058_All	12.92	5.97	-1.11	MYB37	*Arabidopsis thaliana*
Unigene19446_All	7.23	2.57	-1.49	MYB13	*Eutrema salsugineum*
CL5738.Contig1_All	23.08	5.27	-2.13	MYB77	*Eutrema salsugineum*
CL3717.Contig2_All	8.12	1.71	-2.25	MYB12	*Brassica rapa*
Unigene28975_All	2.46	0.01	-7.94	MYB122d	*Arabidopsis lyrata*
CL6419.Contig1_All	6.02	0.01	-9.23	MYB78	*Arabidopsis lyrata*
**CL9137.Contig2_All**	0.01	1.03	6.69	HD-ZIP18	*Arabidopsis thaliana*
Unigene3374_All	0.52	3.1	2.58	HD-ZIP15	*Eutrema salsugineum*
Unigene20784_All	0.74	3.43	2.21	HD-ZIP21	*Arabidopsis thaliana*

CL98.Contig4_All	0.82	3.35	2.03	HD-ZIP17	*Camelina sativa*
CL2896.Contig1_All	2.42	8.16	1.75	HD-ZIP14b	*Eutrema salsugineum*
Unigene5545_All	4.32	14.23	1.72	HD-ZIP6	*Eutrema salsugineum*
CL8168.Contig1_All	2.34	7.36	1.65	bZIP24	*Eutrema salsugineum*
CL5533.Contig2_All	4.82	13.39	1.47	HD-ZIP53	*Eutrema salsugineum*
Unigene8571_All	3.29	8.33	1.34	HD-ZIP23	*Arabidopsis thaliana*
CL3254.Contig1_All	12.89	26.03	1.01	HD-ZIP22	*Camelina sativa*
CL4855.Contig5_All	26.27	10.16	-1.37	HD-ZIP13	*Eutrema salsugineum*
CL4977.Contig3_All	10.92	3.28	-1.74	HD-ZIP5	*Eutrema salsugineum*
CL723.Contig3_All	1.46	0.01	-7.19	HD-ZIP14b	*Eutrema salsugineum*
CL197.Contig3_All	0.05	0.99	4.31	ERF RAF2-7	*Arabidopsis thaliana*
CL5798.Contig2_All	2.81	12.58	2.16	ERF1B	*Eutrema salsugineum*
CL156.Contig5_All	0.74	2.49	1.75	ERF WRI1	*Eutrema salsugineum*
CL1308.Contig3_All	2.71	7.45	1.46	ERF TOE2	*Arabidopsis lyrata*
Unigene2238_All	5.59	14.47	1.37	ERF AIL6	*Eutrema salsugineum*
Unigene5006_All	12.43	25.51	1.04	ERF AIL6	*Eutrema salsugineum*
Unigene9671_All	30.37	12.63	-1.27	ERF13	*Arabidopsis thaliana*
Unigene9911_All	42.35	17.26	-1.29	ERF4a	*Eutrema salsugineum*
Unigene1604_All	353.22	118.74	-1.57	ERF RAP2-3	*Eutrema salsugineum*
CL4895.Contig1_All	37.81	12.6	-1.59	ERF17	*Arabidopsis thaliana*
Unigene12966_All	9.77	2.16	-2.18	ERF4b	*Eutrema salsugineum*
CL1295.Contig8_All	3.25	0.01	-8.34	AP2.2	*Arabidopsis thaliana*
CL4528.Contig2_All	4.96	17.43	1.81	NAC62	*Eutrema salsugineum*
**CL2022.Contig17_All**	0.01	2.43	7.92	bHLH82	*Eutrema salsugineum*
**CL6271.Contig3_All**	0.01	1.61	7.33	bHLH51	*Eutrema salsugineum*
**CL7056.Contig2_All**	0.01	0.78	6.29	bHLH3	*Arabidopsis lyrata*
CL6100.Contig2_All	25.54	12.07	-1.08	bHLH96	*Camelina sativa*
**CL1346.Contig3_All**	0.01	4.53	8.82	MADS-box AGL16	*Eutrema salsugineum*
**CL757.Contig6_All**	0.01	2.29	7.84	MADS-box AGL27	*Arabidopsis thaliana*
**CL1755.Contig6_All**	0.01	1.31	7.03	MADS-box AGL29	*Arabidopsis thaliana*
CL1494.Contig4_All	0.1	6.18	5.95	MADS-box AGL30	*Arabidopsis lyrata*
CL7021.Contig7_All	0.1	1.73	4.11	MADS-box AGL97	*Camelina sativa*
**CL1523.Contig2_All**	0.01	12.81	10.32	ZF C2H2/C2HC	*Camelina sativa*
**CL8710.Contig1_All**	0.01	4.31	8.75	ZF RING	*Arabidopsis thaliana*
**CL898.Contig1_All**	0.01	3.65	8.51	ZF CCHC8a	*Eutrema salsugineum*
**CL1603.Contig7_All**	0.01	3.46	8.43	ZF C3HC4/RINGa	*Eutrema salsugineum*
**CL4465.Contig1_All**	0.01	3.07	8.26	ZF CCHC8b	*Arabidopsis lyrata*
**CL3136.Contig4_All**	0.01	2.89	8.17	ZF BRUTUS	*Eutrema salsugineum*
**CL317.Contig5_All**	0.01	1.34	7.07	ZF RING/FYVE/PHD	*Arabidopsis thaliana*
**CL7159.Contig2_All**	0.01	0.73	6.19	ZF C3HC4/RINGb	*Arabidopsis thaliana*
CL650.Contig3_All	0.64	4.86	2.92	ZF C5HC2	*Arabidopsis thaliana*
CL6521.Contig2_All	1.61	5.45	1.76	ZF CCCH5	*Arabidopsis lyrata*
CL8567.Contig2_All	1.91	6.28	1.72	ZF DOF3.6	*Arabidopsis thaliana*
CL4355.Contig9_All	2.02	5.44	1.43	ZF RING/FYVE/PHD	*Arabidopsis thaliana*
CL458.Contig8_All	5.23	13.86	1.41	ZF CHY/CTCHY/RING	*Arabidopsis thaliana*
CL3278.Contig1_All	2.98	7.2	1.27	ZF RING-H2	*Camelina sativa*
CL2356.Contig1_All	75.7	36.26	-1.06	ZF CCCH29	*Eutrema salsugineum*
CL5646.Contig2_All	11.19	5.32	-1.07	ZF DNL	*Eutrema salsugineum*
Unigene9451_All	78.79	37.11	-1.09	ZF CCCH20	*Eutrema salsugineum*
CL44.Contig2_All	3.77	1.04	-1.86	ZF DOF1.8	*Eutrema salsugineum*
CL993.Contig11_All	1.14	0.31	-1.88	ZF CONSTANS9	*Eutrema salsugineum*
CL650.Contig2_All	4.34	0.18	-4.59	ZF C5HC2	*Arabidopsis thaliana*
Unigene3398_All	2.54	0.09	-4.82	ZF RICESLEEPER2	*Eutrema salsugineum*
CL7776.Contig2_All	1.27	0.01	-6.99	ZF B-box19	*Eutrema salsugineum*
CL93.Contig12_All	5.27	0.04	-7.04	ZF CHY/CTCHY/RING	*Arabidopsis thaliana*
CL9879.Contig1_All	1.65	0.01	-7.37	ZF RING/FYVE/PHD	*Arabidopsis thaliana*
CL403.Contig4_All	4.23	0.01	-8.72	ZF DOF3.7	*Eutrema salsugineum*
CL8003.Contig2_All	4.32	0.01	-8.75	ZF CCCH31	*Brassica oleracea*

CL3676.Contig2_All	9.64	0.01	-9.91	ZF CONSTANS1	*Brassica rapa*
Unigene31435_All	12.95	4.92	-1.40	HSP A-1d	*Brassica napus*

Table S14. Differentially expressed genes (DEGs) related to transcript factors in shoots of *P. cornutum* after 50 mM NaCl treatment for 6 h. C6S RPKM and S6S RPKM indicates the RPKM value of a gene in shoots under control condition for 6 h and salt treatment for 6 h respectively. Fold change equals to $\log_2$(S6S RPKM / C6S RPKM) and indicates the transcript abundance change of each DEGs. Protein is the protein encoded by each DEGs. The Gene ID of each upregulated DEGs with no expression under control condition is in bold.

Gene ID	C6S RPKM	S6S RPKM	Log$_2$ Ratio	Protein	Most homologous species
**CL2762.Contig4_All**	0.01	2.37	7.89	WRKY2	*Arabidopsis thaliana*
**CL10201.Contig4_All**	0.01	0.85	6.41	WRKY27	*Camelina sativa*
CL2623.Contig2_All	1.21	6.1	2.33	WRKY3	*Eutrema salsugineum*
CL2623.Contig7_All	0.41	2.03	2.31	WRKY4	*Eutrema salsugineum*
CL5153.Contig4_All	4.3	17.75	2.05	WRKY33a	*Arabidopsis thaliana*
CL3331.Contig2_All	2.12	6.58	1.63	WRKY19	*Arabidopsis Iyrata*
CL2519.Contig9_All	36.93	17.29	-1.09	WRKY20c	*Arabidopsis Iyrata*
CL6904.Contig1_All	6.84	3.06	-1.16	WRKY62	*Brassica napus*
Unigene13522_All	163.77	68.09	-1.27	WRKY40b	*Arabidopsis Iyrata*
CL4676.Contig4_All	44.86	18.49	-1.28	WRKY46	*Arabidopsis Iyrata*
Unigene12983_All	73.72	27.3	-1.43	WRKY33c	*Eutrema salsugineum*
Unigene13210_All	43.94	15.7	-1.48	WRKY17	*Eutrema salsugineum*
CL6523.Contig4_All	53.34	18.14	-1.56	WRKY18	*Arabidopsis Iyrata*
CL9883.Contig1_All	30.18	9.59	-1.65	WRKY33b	*Eutrema salsugineum*
CL2682.Contig2_All	32.83	8.1	-2.02	WRKY11	*Eutrema salsugineum*
CL731.Contig2_All	7.49	0.9	-3.06	WRKY22b	*Eutrema salsugineum*
Unigene8341_All	5.22	0.56	-3.22	WRKY51	*Arabidopsis Iyrata*
CL731.Contig1_All	35.17	3.45	-3.35	WRKY22a	*Eutrema salsugineum*
CL9252.Contig1_All	2.65	0.22	-3.59	WRKY30	*Eutrema salsugineum*
CL8098.Contig5_All	263.24	21.58	-3.61	WRKY53	*Eutrema salsugineum*
Unigene11700_All	3.04	0.2	-3.93	WRKY49	*Eutrema salsugineum*
Unigene29588_All	21.86	1.16	-4.24	WRKY22c	*Arabidopsis Iyrata*
CL2519.Contig6_All	0.84	0.01	-6.39	WRKY20b	*Arabidopsis thaliana*
CL8143.Contig4_All	5.56	0.01	-9.12	WRKY40a	*Eutrema salsugineum*
**CL6200.Contig1_All**	0.01	1.96	7.61	MYB	*Arabidopsis thaliana*
**CL1196.Contig3_All**	0.01	1.78	7.48	MYB58	*Brassica napus*
**CL9726.Contig1_All**	0.01	1.59	7.31	MYB1	*Capsella rubella*
**CL8144.Contig2_All**	0.01	1.57	7.29	MYB104	*Eutrema salsugineum*
**CL8031.Contig3_All**	0.01	1.54	7.27	MYB106	*Capsella rubella*
**CL2633.Contig9_All**	0.01	1.15	6.85	MYB59	*Arabidopsis thaliana*
CL7166.Contig3_All	1.12	8.08	2.85	MYB	*Arabidopsis Iyrata*
Unigene17362_All	3.71	16.06	2.11	MYB3	*Eutrema salsugineum*
CL3530.Contig3_All	1.72	6.33	1.88	MYB122	*Arabidopsis Iyrata*
CL5993.Contig3_All	1.53	5.39	1.82	MYB2	*Arabidopsis thaliana*
Unigene1381_All	2.57	8.59	1.74	MYB82	*Capsella rubella*
CL8169.Contig5_All	0.52	1.59	1.61	MYB3R-1	*Eutrema salsugineum*
Unigene11649_All	41.66	96.58	1.21	MYB28	*Eutrema salsugineum*
CL577.Contig2_All	2.48	5.54	1.16	MYB20	*Eutrema salsugineum*
CL6200.Contig3_All	3.32	7.19	1.11	MYB	*Arabidopsis Iyrata*
CL5738.Contig5_All	99.98	42.72	-1.23	MYB44	*Eutrema salsugineum*
CL4647.Contig1_All	81.68	28.88	-1.50	MYB34	*Brassica napus*
CL9574.Contig2_All	2.94	0.38	-2.95	MYB15	*Eutrema salsugineum*
Unigene14726_All	0.12	1.88	3.97	HD-ZIP10	*Arabidopsis thaliana*
CL4855.Contig1_All	0.32	2.24	2.81	HD-ZIP13	*Eutrema salsugineum*
Unigene6802_All	0.67	3.76	2.49	HD-ZIP7	*Eutrema salsugineum*
CL1078.Contig5_All	2.01	8.48	2.08	HD-ZIP HDG1	*Brassica rapa*

Unigene199_All	12.39	47.22	1.93	HD-ZIP16	*Eutrema salsugineum*
CL791.Contig3_All	13.47	5.73	-1.23	bZIP2	*Brassica rapa*
CL5737.Contig1_All	3.79	1.21	-1.66	bZIP7	*Eutrema salsugineum*
Unigene13416_All	67.26	26.68	-1.33	ERF1Ab	*Arabidopsis Iyrata*
Unigene1753_All	66.54	22.36	-1.57	ERF1Aa	*Eutrema salsugineum*
CL4895.Contig1_All	183.13	59.19	-1.63	ERF17	*Eutrema salsugineum*
CL6308.Contig2_All	2.9	0.89	-1.70	AP2	*Arabidopsis thaliana*
Unigene12966_All	10.53	3.17	-1.73	ERF4b	*Eutrema salsugineum*
Unigene9911_All	71.05	17.69	-2.01	ERF4a	*Eutrema salsugineum*
Unigene9671_All	46.81	10.36	-2.18	ERF13	*Arabidopsis thaliana*
Unigene8212_All	8.98	2.03	-2.15	ERF6	*Brassica napus*
Unigene13757_All	5.2	0.8	-2.70	ERF4c	*Eutrema salsugineum*
Unigene8938_All	2.06	0.01	-7.69	ERF1	*Eutrema salsugineum*
CL1295.Contig8_All	3.77	0.01	-8.56	AP2.2	*Arabidopsis thaliana*
**CL4528.Contig2_All**	0.01	10.39	10.02	NAC62	*Eutrema salsugineum*
CL9194.Contig1_All	1.5	12.31	3.04	bHLH92	*Arabidopsis thaliana*
CL1277.Contig6_All	4.82	10.61	1.14	bHLH67	*Arabidopsis thaliana*
CL7979.Contig1_All	2.32	0.48	-2.27	bHLH121	*Arabidopsis thaliana*
**CL1494.Contig4_All**	0.01	6.86	9.42	MADS-box30	*Arabidopsis Iyrata*
CL757.Contig4_All	2.55	11.74	2.20	MADS-box31	*Arabidopsis thaliana*
**CL93.Contig7_All**	0.01	10.87	10.09	ZF CHY/CTCHY/RING	*Eutrema salsugineum*
**CL9879.Contig1_All**	0.01	4.86	8.92	ZF RING/FYVE/PHD	*Arabidopsis thaliana*
**CL3136.Contig4_All**	0.01	4.32	8.75	ZF BRUTUS	*Eutrema salsugineum*
**CL898.Contig1_All**	0.01	3.84	8.58	ZF CCHC	*Eutrema salsugineum*
**CL3136.Contig3_All**	0.01	3.39	8.41	ZF BRUTUS	*Eutrema salsugineum*
**CL993.Contig10_All**	0.01	2.01	7.65	ZF CONSTANS9	*Eutrema salsugineum*
**CL5377.Contig2_All**	0.01	0.96	6.58	ZF BBX27	*Eutrema salsugineum*
CL993.Contig9_All	3.68	30.72	3.06	ZF CONSTANS9	*Eutrema salsugineum*
CL8528.Contig2_All	3.55	27.83	2.97	ZF CONSTANS6	*Arabidopsis Iyrata*
CL4355.Contig9_All	0.34	2.36	2.80	ZF RING/FYVE/PHD	*Arabidopsis thaliana*
CL4905.Contig1_All	0.31	1.61	2.38	ZF BBX21	*Eutrema salsugineum*
CL44.Contig2_All	0.46	2.12	2.20	ZF DOF1.8	*Eutrema salsugineum*
CL993.Contig2_All	0.62	2.65	2.10	ZF CONSTANS1	*Camelina sativa*
CL242.Contig3_All	0.66	2.61	1.98	ZF7	*Eutrema salsugineum*
CL2491.Contig3_All	0.95	3.59	1.92	ZF CCHC10	*Eutrema salsugineum*
Unigene1924_All	0.84	3.11	1.89	ZF NURCRACKER	*Eutrema salsugineum*
CL4287.Contig2_All	7.84	28.43	1.86	ZF DOF2.2	*Capsella rubella*
CL1212.Contig1_All	43.84	139.08	1.67	ZF CONSTANS16	*Arabidopsis Iyrata*
Unigene30519_All	28.79	85.53	1.57	ZF CONSTANS6	*Brassica nupus*
CL5510.Contig2_All	2.92	8.65	1.57	ZF DOF2.2	*Arabidopsis Iyrata*
CL2415.Contig1_All	38.34	110.79	1.53	ZF CONSTANS9	*Brassica nupus*
Unigene5109_All	3.58	9.97	1.48	ZF DOF2.2	*Eutrema salsugineum*
CL7040.Contig2_All	9.47	22.69	1.26	ZF Cys2/His2 2	*Eutrema salsugineum*
CL1518.Contig1_All	1.92	4.55	1.24	ZF GATA	*Eutrema salsugineum*
CL7040.Contig1_All	37.54	79.52	1.08	ZF Cys2/His2 2	*Eutrema salsugineum*
Unigene30518_All	33.6	70.07	1.06	ZF CONSTANS6	*Camelina sativa*
Unigene30520_All	14.23	6.83	-1.06	ZF CONSTANS7	*Camelina sativa*
CL4355.Contig2_All	2.94	1.37	-1.10	ZF RING/FYVE/PHD	*Arabidopsis thaliana*
CL8271.Contig4_All	38.12	17.23	-1.15	ZF AN1/C2H2 13	*Arabidopsis Iyrata*
CL5396.Contig1_All	24.59	11.04	-1.16	ZF RING/FYVE/PHD	*Arabidopsis thaliana*
CL7279.Contig2_All	3.19	1.39	-1.20	ZF C3HC4/RING	*Arabidopsis thaliana*
CL8271.Contig1_All	59.5	21.89	-1.44	ZF AN1/C2H2 13	*Arabidopsis Iyrata*
CL458.Contig8_All	2.75	0.88	-1.64	ZF CHY/CTCHY/RING	*Arabidopsis thaliana*
CL1603.Contig1_All	3	0.95	-1.66	ZF C3HC4/RING	*Arabidopsis thaliana*
Unigene2658_All	6.46	1.93	-1.74	ZF CCHC20	*Brassica rapa*
Unigene13138_All	1.93	0.33	-2.55	ZF BBX28	*Arabidopsis thaliana*
CL1518.Contig4_All	1.44	0.23	-2.65	ZF GATA27	*Arabidopsis Iyrata*
CL4465.Contig1_All	1.01	0.01	-6.66	ZF CCHC8	*Camelina sativa*

CL2076.Contig5_All	1.51	0.01	-7.24	ZF A20/AN1	*Camelina sativa*
CL2491.Contig1_All	1.65	0.01	-7.37	ZF CCHC10	*Eutrema salsugineum*
**Unigene16812_All**	0.01	7.8	9.61	HSP3	*Arabidopsis thaliana*
**CL1608.Contig1_All**	0.01	6.48	9.34	HSP6	*Eutrema salsugineum*
**Unigene329_All**	0.01	2.39	7.90	HSP A-4a	*Arabidopsis Iyrata*
Unigene7975_All	1.07	38.2	5.16	HSP21	*Arabidopsis Iyrata*
Unigene3542_All	1.65	8.42	2.35	HSP23.6	*Arabidopsis thaliana*
Unigene6938_All	6.48	19.49	1.59	HSP2	*Arabidopsis thaliana*
CL2361.Contig5_All	6.91	13.98	1.02	HSP70	*Arabidopsis thaliana*
CL4377.Contig3_All	5.28	1.98	-1.42	HSP89.1	*Arabidopsis Iyrata*
Unigene1831_All	37.58	12.93	-1.54	HSP A-4a	*Arabidopsis Iyrata*
CL4153.Contig2_All	38.05	12.03	-1.66	HSP A-4a	*Arabidopsis Iyrata*

Table S15. Expression pattern validation of 20 randomly selected genes in roots of *P. cornutum* under 50 mM NaCl treatment for 6 and 24 h by qRT-PCR method.

**Gene ID in Root**	**6 h**		**24 h**	
	**RNA-seq**	**Real-time PCR**	**RNA-seq**	**Real-time PCR**
**CL110.Contig1_All**	10.2	9.08±0.25	10.36	9.94±0.29
**CL8902.Contig1_All**	9.82	9.33±0.34	9.75	9.98±0.47
**CL1547.Contig7_All**	8.75	7.56±0.65	2.84	2.78±0.04
**CL4893.Contig2_All**	8.3	7.75±0.48	7.55	4.90±0.16
**CL1814.Contig2_All**	6.9	4.88±0.43	7.55	6.85±0.18
**Unigene29368_All**	1.73	2.22±0.24	9.45	8.74±0.21
**Unigene16702_All**	1.69	2.01±0.18	1.26	1.38±0.02
**Unigene2556_All**	1.58	1.24±0.31	1.07	-1.25±0.12
**Unigene10031_All**	1.24	1.99±0.23	1.3	1.57±0.14
**CL180.Contig7_All**	5.73	2.66±0.51	5.17	4.60±0.22
**CL5558.Contig8_All**	2.88	3.01±0.16	1.51	1.63±0.16
**CL8105.Contig1_All**	2.5	2.48±0.14	2.11	1.97±0.18
**CL1918.Contig4_All**	2.22	2.20±0.21	2.2	2.73±0.08
**CL5798.Contig2_All**	2.16	1.99±0.15	9.53	8.54±0.27
**CL5176.Contig7_All**	1.84	1.97±0.17	8.43	3.42±0.19
**CL2131.Contig2_All**	-10.27	-4.93±0.42	-4.29	-5.34±0.18
**CL2682.Contig2_All**	-1.09	-1.22±0.0.13	-1.75	-1.83±0.13
**CL2337.Contig2_All**	-1.51	-1.62±0.24	-1.53	-1.40±0.09
**CL1301.Contig6_All**	-1.63	-1.59±0.22	-2.05	-2.63±0.16
**Unigene6631_All**	-1.66	-1.59±0.17	-2.19	-1.94±0.02

Table S16. Expression pattern validation of 20 randomly selected genes in shoots of *P. cornutum* under 50 mM NaCl treatment for 6 and 24 h by qRT-PCR method.

**Gene ID in Shoot**	**6 h**		**24 h**	
	**RNA-seq**	**Real-time PCR**	**RNA-seq**	**Real-time PCR**
**CL1608.Contig1_All**	9.34	12.52±1.97	2.06	2.47±0.19
**CL1586.Contig2_All**	8.81	3.28±0.24	-2.12	1.06±0.08
**CL2.Contig46_All**	7.91	2.87±0.22	5.55	3.60±0.08
**CL3679.Contig2_All**	7.44	7.23±0.13	7.53	6.46±0.23
**Unigene8549_All**	1.25	1.37±0.15	-1.74	-2.12±0.04
**Unigene7975_All**	3.13	3.46±0.15	5.16	1.66±0.06
**CL3117.Contig24_All**	4.61	4.40±0.25	5.98	4.91±0.07
**CL3756.Contig11_All**	2.58	2.89±0.13	7.81	3.89±0.22
**CL338.Contig5_All**	2.5	2.51±0.19	1.74	1.48±0.09
**CL2477.Contig4_All**	2.42	2.34±0.06	1.18	1.21±0.15
**Unigene1617_All**	2.38	2.43±0.37	2.29	2.66±0.19
**CL1825.Contig7_All**	1.75	1.64±0.04	1.92	1.88±0.17

**CL1767.Contig3_All**	1.64	1.56±0.08	1.36	1.48±0.24
**CL5344.Contig1_All**	1.19	1.41±0.15	1.55	1.72±0.16
**CL7594.Contig2_All**	-4.22	-3.77±0.13	-3.75	-3.45±0.07
**CL731.Contig1_All**	-3.35	-2.32±0.19	-1.94	-1.89±0.09
**Unigene3186_All**	-3.1	-1.36±0.05	-1.8	-2.09±0.14
**Unigene29635_All**	-2.82	-2.48±0.05	-2.53	-2.52±0.21
**CL7117.Contig1_All**	-2.58	-2.03±0.13	-1.59	-1.24±0.08
**CL1590.Contig1_All**	-2.04	-2.26±0.08	-4.02	-3.71±0.07

17
